# Estimating global and regional between-country inequality in routine childhood vaccine coverage in 195 countries and territories from 2019 to 2021: a longitudinal study

**DOI:** 10.1016/j.eclinm.2023.102042

**Published:** 2023-06-08

**Authors:** Xiaozhen Lai, Haijun Zhang, Koen B. Pouwels, Bryan Patenaude, Mark Jit, Hai Fang

**Affiliations:** a Department of Health Policy and Management, School of Public Health, Peking University, Beijing, China; b Health Economics Research Centre, Nuffield Department of Population Health, University of Oxford, Oxford, UK; c Department of International Health, Johns Hopkins Bloomberg School of Public Health, Baltimore, USA; d International Vaccine Access Center, Johns Hopkins Bloomberg School of Public Health, Baltimore, USA; e Department of Infectious Disease Epidemiology, Faculty of Epidemiology and Population Health, London School of Hygiene and Tropical Medicine, London, UK; f Centre for Mathematical Modelling of Infectious Diseases, London School of Hygiene and Tropical Medicine, London, UK; g School of Public Health, University of Hong Kong, Hong Kong SAR, China; h China Center for Health Development Studies, Peking University, Beijing, China; i Peking University Health Science Center-Chinese Center for Disease Control and Prevention Joint Research Center for Vaccine Economics, Peking University, Beijing, China

**Keywords:** Inequality, Vaccine coverage, COVID-19 pandemic, Global child health

## Abstract

**Background:**

Global routine childhood vaccine coverage has plateaued in recent years, and the COVID-19 pandemic further disrupted immunisation services. We estimated global and regional inequality of routine childhood vaccine coverage from 2019 to 2021, particularly assessing the impacts of the COVID-19 pandemic.

**Methods:**

We used longitudinal data for 11 routine childhood vaccines from the WHO-UNICEF Estimates of National Immunization Coverage (WUENIC), including 195 countries and territories in 2019–2021. The slope index of inequality (SII) and relative index of inequality (RII) of each vaccine were calculated through linear regression to express the difference in coverage between the top and bottom 20% of countries at the global and regional levels. We also explored inequalities of routine childhood vaccine coverage by WHO regions and unvaccinated children by income groups.

**Findings:**

Globally between January 1, 2019 and December 31, 2021, most childhood vaccines showed a declining trend in coverage, and therefore an increasing number of unvaccinated children, especially in low-income and lower-middle-income countries. Between-country inequalities existed for all 11 routine childhood vaccine coverage indicators. The SII for the third dose of diphtheria-tetanus-pertussis-containing vaccine (DTP3) coverage was 20.1 percentage points (95% confidence interval: 13.7, 26.5) in 2019, and rose to 23.6 (17.5, 30.0) in 2020 and 26.9 (20.0, 33.8) in 2021. Similar patterns were found for RII results and in other routine vaccines. In 2021, the second dose of measles-containing vaccine (MCV2) coverage had the highest global absolute inequality (31.2, [21.5–40.8]), and completed rotavirus vaccine (RotaC) coverage had the lowest (7.8, [–3.9, 19.5]). Among six WHO regions, the European Region consistently had the lowest inequalities, and the Western Pacific Region had the largest inequalities for many indicators, although both increased from 2019 to 2021.

**Interpretation:**

Global and regional inequalities of routine childhood vaccine coverage persisted and substantially increased from 2019 to 2021. These findings reveal economic-related inequalities by vaccines, regions, and countries, and underscore the importance of reducing such inequalities. These inequalities were widened during the COVID-19 pandemic, resulting in even lower coverage and more unvaccinated children in low-income countries.

**Funding:**

10.13039/100000865Bill & Melinda Gates Foundation.

**Research in context**

**Evidence before this study**

There is a growing need to reduce vaccine inequalities across countries, in line with the WHO-initiated Immunization Agenda 2030 (IA2030). Previous studies have found significant decreases in the coverage of some childhood vaccines globally or in specific countries during the COVID-19 pandemic, and some studies raised issues concerning the increased inequalities after the COVID-19 pandemic. However, previous studies mainly focused on the third dose of diphtheria-tetanus-pertussis-containing vaccine (DTP3) and the first dose of measles-containing vaccine (MCV1), or were conducted in a single country, and none of them formally analysed and reported the inequalities from a global perspective. Vaccination programmes in low-income countries and regions were more severely disrupted by the COVID-19 pandemic, and vaccination inequalities need to be further analysed across countries.

**Added value of this study**

This study revealed that global and regional routine childhood vaccine coverage declined from 2019 to 2020, and further fell in 2021, so global vaccination programmes in 2021 did not recover from the COVID-19 pandemic. In terms of inequalities, between-country inequalities existed in all 11 routine childhood vaccination coverage. It was also found that the inequalities of routine childhood vaccine coverage substantially increased from 2019 to 2020, and further rose in 2021 with much larger gaps between high- and low-income countries. Within different WHO regions, the inequalities were diverse and varied substantially from 2019 to 2021, with the European Region consistently having the lowest inequalities, and the Western Pacific Region having the largest inequalities for many indicators.

**Implications of all the available evidence**

The COVID-19 pandemic has reduced global, regional, and national routine childhood vaccine coverage to the lowest levels of the past decade, and the recovery of immunisation services to pre-pandemic levels has not been achieved as of December 2021. The inequalities in routine immunisation coverage between high- and low-income countries have significantly widened at the global and regional levels. The less resilient livelihood and health systems of low-income countries pose a significant risk of increased vulnerability of children living in these areas to vaccine-preventable diseases in the near future; thus amplifying the burden on health systems. The global community and country health systems must prioritise the reinforcement of primary health care systems and vaccine programmes in low-income and middle-income countries to mitigate unfavourable outcomes and address gaps in routine childhood vaccination coverage.

## Introduction

Vaccination is one of the most effective interventions against infectious diseases which has averted millions of deaths worldwide.^1^^,^^2^ Although global and regional coverage of routine childhood vaccines has increased since 1980, inequalities have persisted across countries with high-income countries usually having much higher routine childhood vaccine coverage than their low-income counterparts.^3^, ^4^, ^5^, ^6^, ^7^, ^8^ Reducing inequalities and ensuring no one is left behind are integral to achieving the United Nations Sustainable Development Goals, and inequalities in vaccination coverage among countries remain a topic worthy of continued attention.

The COVID-19 pandemic severely disrupted global, regional and national routine immunisation services in 2020.^4^ Modelled estimates suggested more than 8 million children in 2020 missed out the third dose of diphtheria-tetanus-pertussis-containing vaccine (DTP3) and the first dose of measles-containing vaccine (MCV1) owing to the COVID-19 pandemic.^9^ It was noticed that in 2020, the impacts of the pandemic on immunisation services varied by World Health Organization (WHO) regions.^10^^,^^11^ Until now, however, studies on this topic mainly focused on DTP3 and MCV1, or were conducted in a single country,^12^ and whether global immunisation programmes recovered in 2021 remained unknown.

Additionally, as a key factor for Global Vaccine Action Plan 2020,^4^^,^^5^ equity has been regarded as the foundation of Immunization Agenda 2030 (IA2030) with a vision of “A world where everyone, everywhere, at every age, fully benefits from vaccines for good health and well-being”.^13^^,^^14^ Inequitable distribution is not an issue unique to COVID-19 vaccines as mentioned by the WHO,^15^^,^^16^ and it is urgent to know the impacts of COVID-19 pandemic on global and regional between-country inequalities of routine childhood vaccines, since the inequalities of routine childhood vaccine coverage in recent years would influence the morbidities and mortalities of vaccine-preventable diseases, especially after the relaxation of COVID-19 prevention and control measures.^17^ Low-income countries usually faced much more inequitable access to other routine vaccines, which could be even worse during the COVID-19 pandemic. Although some studies raised issues concerning the increased inequalities after the COVID-19 pandemic, none of them formally analysed and reported them from a global perspective. It is important to explore childhood vaccination coverage inequalities facing the COVID-19 pandemic and identify more severely affected countries and regions. In this case, this study estimated global and regional inequalities of routine childhood vaccine coverage from 2019 to 2021, with a focus on the impacts of the COVID-19 pandemic.

## Methods

### Data source

We used WHO-UNICEF Estimates of National Immunization Coverage (WUENIC) to analyse global and regional cross-country inequalities of immunisation programme services from 2019 to 2021.^18^^,^^19^ The estimates were based on data officially reported to WHO and UNICEF by Member States, and supplemented by survey results in published and grey literature following the methods that have been endorsed by external advisory groups.^18^ The WUENIC provides the world's largest aggregated dataset on immunisation trends annually across 195 WHO and UNICEF Member States, with the most updated estimates in 2021 released in July 2022. Coverage figures for 11 routine childhood vaccines in 195 countries and territories in 2019, 2020 and 2021 were analysed in the present study, including the first dose of Bacillus Calmette-Guérin vaccine (BCG), the first dose of DTP vaccine (DTP1), the third dose of DTP vaccine (DTP3), the third dose of Polio vaccine (Pol3), the first dose of rubella-containing vaccine (RCV1), the first dose of MCV (MCV1), the second dose of MCV (MCV2), the third dose of Hepatitis B vaccine (HepB3), the third dose of *Haemophilus influenzae type b* vaccine (Hib3), the third dose of pneumococcal conjugate vaccine (PCV3), and completed rotavirus series, two or three doses (RotaC).

The study only included countries and territories with available data in all three years for each vaccine type. Childhood vaccine coverage was defined as the proportion of children in one country or territory who received at least the stated vaccine dose in their first year of life. To assess economic-related inequalities from 2019 to 2021, countries and territories were ranked by their annual GDP per capita from World Bank.^20^ The World Bank categorised 195 countries and territories into four income groups: low-income (n = 28), lower-middle-income (n = 55), upper-middle-income (n = 52), and high-income (n = 60) in 2019. The population size of children under one-year-old was obtained from the United Nations Department of Economics and Social Affairs, Population Division.^21^ Regional analyses were based on six WHO regions of Africa, Americas, Eastern Mediterranean, Europe, South-East Asia, and Western Pacific.

### Ethics

The study utilised secondary, de-identified aggregated data and did not involve any primary data collection or direct interactions with human participants. Therefore, no ethical approval was obtained for this study.

### Statistical analysis

Routine childhood vaccine coverage estimates were weighted by the annual number of children younger than one year in each country to estimate global and regional annual coverage in countries with available data in 2019–2021, and the year-by-year trend was displayed in line figures. The number of children who did not receive each type of routine childhood vaccine in countries with available data was reported at the global and four income group levels, by multiplying the estimated unvaccinated proportion and eligible population size.

For inequalities of routine childhood vaccination coverage, countries and territories in each year were ranked by GDP per capita and disaggregated into 5 quintiles. The slope index of inequality (SII) and relative index of inequality (RII) of each vaccine were calculated through linear regression. The SII is an indicator of absolute inequality representing the beta statistic of the regression between weighted coverage and wealth quintiles, and the RII is an indicator of relative inequality closely related to the SII.^22^^,^^23^ These indicators can be interpreted as the regression-based difference (SII) or ratio (RII) between the highest and lowest extremes of wealth distribution. A positive SII or RII larger than one manifested higher coverage with increasing wealth, and it was recommended that both absolute and relative measures should be presented when describing inequalities.^22^, ^23^, ^24^ The Jonckeere–Terpstra test and Willcoxon-Mann-Whitney rank sum test were used to test the trends of inequalities. The Jonckeere–Terpstra test examined SIIs and RIIs from 2019 to 2021 at global and regional levels, and the Willcoxon-Mann-Whitney rank sum test compared SIIs and RIIs in 2019 and 2021 after excluding data in 2020. A Bartlett's test was performed prior to the Jonckeere–Terpstra test, and all p > 0.05 at global and regional levels indicated the acceptance of the null, i.e., same variability across groups. It was noted that WUENIC provides data in fewer countries and territories than the Global Burden of Disease (GBD) study, while the GBD has coverage estimates for all routine childhood vaccines in 204 countries and territories only from 1980 to 2019, making it hard to access the impact of the COVID-19 pandemic.^7^ Therefore, the inequalities of routine childhood vaccine coverage in 2019 were also estimated using the GBD data and compared with those using WUENIC data as a sensitivity analysis, although WUENIC and GBD estimates were very similar and consistent.^13^

Additionally, the number and proportion of countries and territories with lower routine childhood vaccine coverage in 2021 than in 2019 were counted to determine the most severely impacted income groups. We then took DTP3 vaccination status as an example to map the countries and territories with the largest declines in DTP3 vaccination coverage from 2019 to 2021, and with the largest number of unvaccinated children. All data processing and analysing were done in Stata 17 software. To compute 95% confidence intervals (CIs) for global and regional vaccine coverage inequalities, we sampled 1000 random draws and took the ordinal 2.5th and 97.5th percentile of draws.

### Role of the funding source

The funder of this study had no role in study design, data collection, data analysis, data interpretation, or writing and submitting this manuscript.

## Results

### Global and regional routine childhood vaccination coverage from 2019 to 2021

**Fig. 1:**
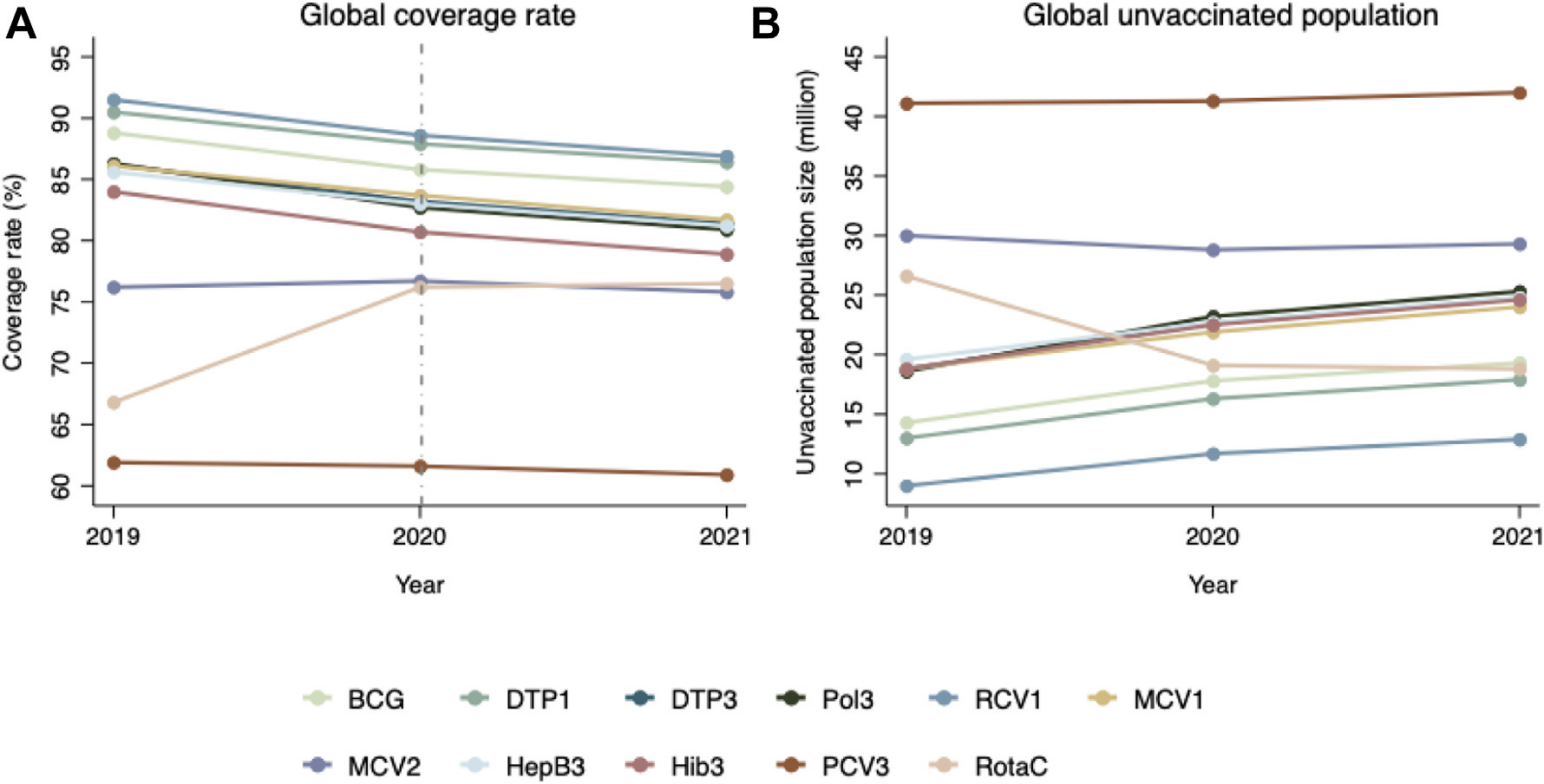
**Global weighted coverage and unvaccinated population for 11 routine childhood vaccines from 2019 to 2021.** Notes: BCG, Bacillus Calmette-Guérin vaccine; DTP, Diphtheria-tetanus-pertussis vaccine; Pol, Polio vaccine; RCV, Rubella-containing vaccine; MCV, Measles-containing vaccine; HepB, Hepatitis B vaccine; Hib, *Haemophilus influenzae* type b vaccine; PCV, pneumococcal conjugate vaccine, RotaC, Completed rotavirus vaccine.

After weighted by country-level population size, globally all childhood vaccination coverage indicators showed a declining trend from 2019 to 2021 except for RotaC (Fig. 1A). For example, global DTP3 coverage decreased from 86.2% (81.2, 91.3) in 2019 to 83.2% (78.2, 88.2) in 2020 and 81.4% (76.1, 86.7) in 2021 (webappendix Table S1). Global MCV1 coverage decreased from 86.1% (80.4, 91.9) in 2019 to 83.7% (78.5, 88.9) in 2020 and 81.7% (75.9, 87.5) in 2021. PCV3 coverage slightly fell from 61.9% (40.5, 83.4) in 2019 to 61.6% (43.0–80.3) in 2020 and 60.9% (44.3, 77.5) in 2021. Among 11 routine childhood vaccines, Pol3 coverage had the largest global decline from 86.3% in 2019 to 80.9% (75.3, 86.4) in 2021. The declines in vaccine coverage in 2019–2021 were not found to be a continuation of the previous trend in 2010–2019 (webappendix Table S1).

**Fig. 2:**
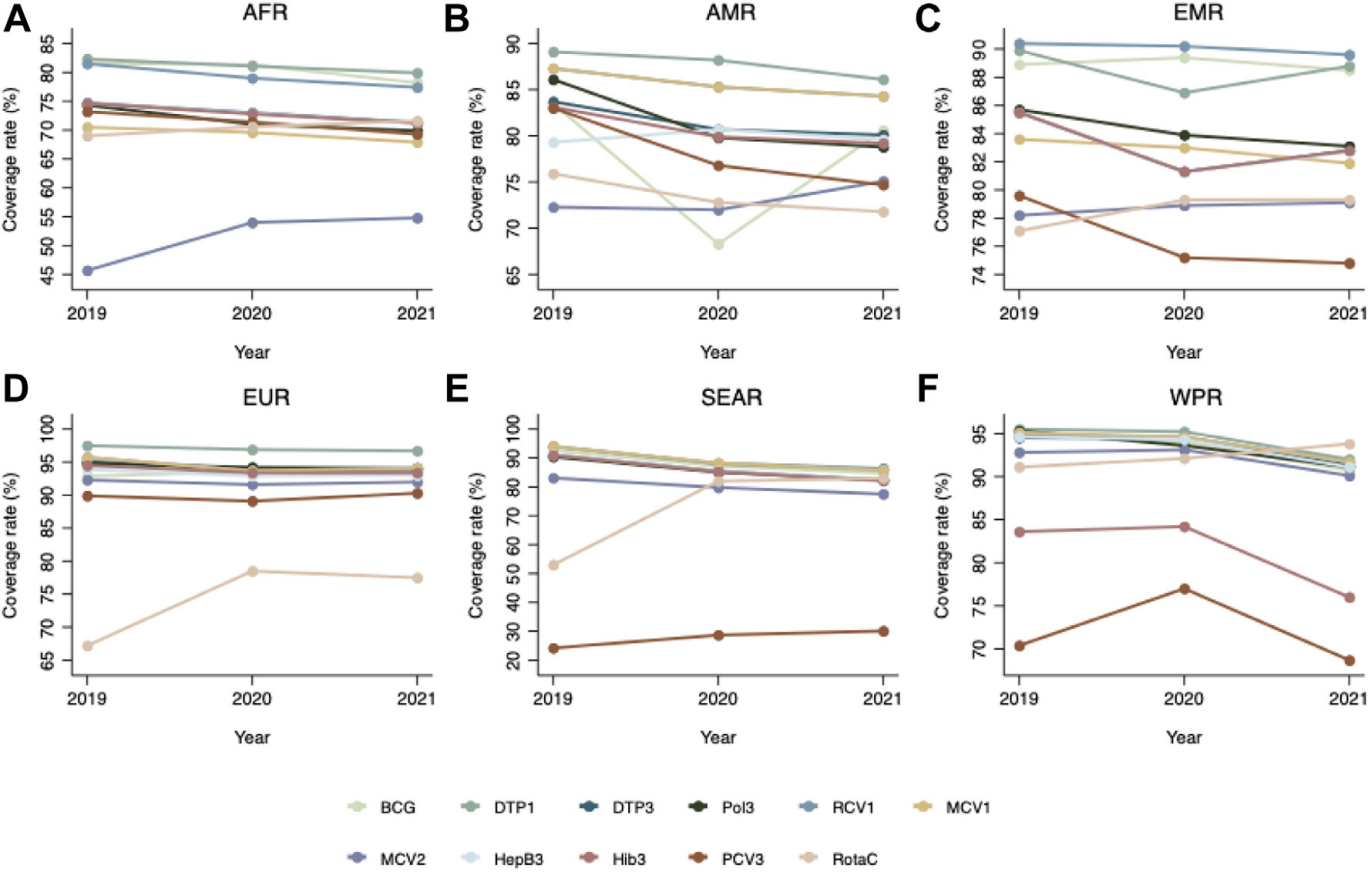
**Coverage for 11 routine childhood vaccines from 2019 to 2021 in six WHO regions.** Notes: AFR, the African Region; AMR, the Region of the Americas; EMR, the Eastern Mediterranean Region; EUR, the European Region; SEAR, the South-East Asian Region; WPR, the Western Pacific Region; BCG, Bacillus Calmette-Guérin vaccine; DTP, Diphtheria-tetanus-pertussis vaccine; Pol, Polio vaccine; RCV, Rubella-containing vaccine; MCV, Measles-containing vaccine; HepB, Hepatitis B vaccine; Hib, *Haemophilus influenzae* type b vaccine; PCV, Pneumococcal conjugate vaccine, RotaC, Completed rotavirus vaccine.

Regional routine childhood vaccination coverage indicators basically followed the same declining pattern, with variations across WHO regions (Fig. 2). For all 11 routine childhood vaccines, the European Region usually had the highest coverage, while the African Region had the lowest. The South-East Asian Region had the largest decrease in routine childhood vaccination coverage during the COVID-19 pandemic in 2020 and 2021, and only the European Region stopped further declines from 2020 to 2021. For instance, DTP3 coverage in the European Region decreased from 95.1% (93.4, 96.9) in 2019 to 93.8% (91.9, 95.8) in 2020 and 93.9% (92.1, 95.6) in 2021 (webappendix Table S2), and that in the African Region decreased from 74.7% (66.2, 83.3) in 2019 to 73.0% (65.3, 80.8) in 2020 and 71.3% (63.7, 78.9) in 2021. The regional difference in DTP3 coverage between Europe and Africa was enlarged from 20.4% in 2019 to 22.6% in 2021. In addition, among six WHO regions, the South-East Asian Region had the largest decrease in DTP3 vaccination coverage in two years, from 91.1% (88.7, 93.5) in 2019 to 85.5% (81.7, 89.4) in 2020 and 82.4% (74.6, 90.3) in 2021. PCV3 coverage in 2021 had the largest difference among 6 WHO regions with 90.2% (86.8, 93.6) in Europe and 30.1% (6.1, 54.1) in South-East Asia.

Fig. 1B reported the increasing number of unvaccinated children for each vaccine type from 2019 to 2021 except for MCV2 and RotaC. For example, there were 18.8 million children worldwide not completing DTP3 vaccination in 2019, and this number increased to 22.6 million in 2020 and 24.7 million in 2021 (Table 1). When dividing them into different income groups, more than 50% of these unvaccinated children were from lower-middle-income countries. By contrast, high-income countries had a fairly small number of unvaccinated children. The number also demonstrated an increasing trend from 2019 to 2021 in low-income and upper-middle-income countries.

**Table 1:** Unvaccinated population for 11 routine childhood vaccines from 2019 to 2021 in country income groups (million children).

Vaccine type	Global			Low income countries			Lower-middle income countries			Upper-middle income countries			High income countries		
	2019	2020	2021	2019	2020	2021	2019	2020	2021	2019	2020	2021	2019	2020	2021
BCG (n = 157)	14.3	17.8	19.3	4.4	4.5	5.7	7.2	9.5	11.1	2.1	3.5	2.2	0.6	0.3	0.3
DTP1 (n = 195)	13.0	16.3	17.9	3.7	4.1	4.8	7.2	10.0	10.7	1.8	1.9	2.1	0.3	0.3	0.3
DTP3 (n = 195)	18.8	22.6	24.7	5.3	5.8	6.8	10.4	13.1	14.2	2.4	2.9	2.9	0.7	0.8	0.8
Pol3 (n = 195)	18.6	23.2	25.3	5.2	5.8	6.8	10.7	13.6	14.7	2.0	3.0	3.0	0.7	0.8	0.8
RCV1 (n = 171)	9.0	11.7	12.9	0.9	1.0	1.0	5.6	7.9	9.1	1.7	1.9	2.0	0.8	0.9	0.8
MCV1 (n = 195)	18.8	21.9	24.0	6.4	6.8	7.7	9.6	11.8	13.2	2.0	2.4	2.3	0.8	0.9	0.8
MCV2 (n = 177)	30.0	28.8	29.3	6.4	6.2	6.3	18.4	17.6	18.5	4.2	4.0	3.5	1.0	1.0	1.0
HepB3 (n = 190)	19.6	22.7	24.8	5.3	5.8	6.8	10.5	13.1	14.2	2.9	2.8	2.8	0.9	1.0	1.0
Hib3 (n = 192)	18.8	22.5	24.6	5.3	5.8	6.8	10.5	13.1	14.2	2.2	2.7	2.7	0.8	0.9	0.9
PCV3 (n = 144)	41.1	41.3	42.0	4.4	4.9	5.7	33.0	31.4	31.2	2.6	3.5	3.6	1.1	1.5	1.5
RotaC (n = 102)	26.6	19.1	18.8	6.8	6.1	5.9	14.8	8.2	8.1	3.1	3.0	3.1	1.9	1.8	1.7

Notes: BCG, Bacillus Calmette-Guérin vaccine; DTP, Diphtheria-tetanus-pertussis vaccine; Pol, Polio vaccine; RCV1, Rubella-containing vaccine; MCV, Measles-containing vaccine; HepB, Hepatitis B vaccine; Hib, *Haemophilus influenzae type b* vaccine; PCV, Pneumococcal conjugate vaccine, RotaC, Completed rotavirus vaccine.

### Global and regional inequalities from 2019 to 2021

Between-country inequalities were found in the coverage of all 11 routine childhood vaccines, as countries with higher GDP per capita usually had higher routine childhood vaccination coverage than those with lower GDP per capita. In 2019, there existed large inequalities in routine childhood vaccination coverage, which became even larger during the COVID-19 pandemic (Table 2). The absolute difference in coverage between the top and bottom of the wealth distribution (SII) for DTP3 vaccine coverage in 195 countries and territories was 20.1 percentage points (95% CI: 13.7, 26.5) in 2019, and rose to 23.6 percentage points (17.5, 30.0) in 2020 and 26.9 percentage points (20.0, 33.8) in 2021. Similarly, the SII for MCV1 coverage increased from 23.7 percentage points (17.1, 30.4) in 2019 to 25.5 percentage points (19.1, 32.0) in 2020 and 30.0 percentage points (23.0, 37.0) in 2021. The global PCV3 inequality increased from 17.2 percentage points (8.9, 25.5) in 2019 to 19.8 percentage points (11.8, 27.8) in 2020, and 24.4 percentage points (15.8, 33.1) in 2021, although the global PCV3 coverage did not change much in 2019–2021. From 2019 to 2021, MCV2 coverage had the highest global absolute inequality (31.2 percentage points, [21.5–40.8]), and RotaC coverage had the lowest (7.8 percentage points, [−3.9, 19.5]). The results of RII were consistent with those of SII, since they were closely related. Taking DTP3 vaccine coverage as an example, in relative terms, children in countries at the top of the wealth distribution were 1.25 (1.16, 1.35) times more likely to be vaccinated with DTP3 in 2019 than those in countries at the bottom of the wealth distribution, and the RII increased to 1.32 (1.22, 1.41) in 2020 and 1.37 (1.25, 1.49) in 2021. Statistical tests indicated that the increased inequality from 2019 to 2021 was significant globally (Table 3), and it was not a continuation of the trend in 2010–2018 (Table 4). The overall conclusions were robust if we kept all countries and territories with available data in 2019–2021 (webappendix Table S3), adopted the GBD vaccine coverage data in 2019 (webappendix Table S4), or used different socioeconomic grouping strategies including 4 quartiles, 10 deciles, and 4 income groups (webappendix Table S5).

**Table 2:** Global slope index of inequality and relative index of inequality for 11 routine childhood vaccine coverage from 2019 to 2021.

Vaccine type	Slope index of inequality (SII)^a^			Relative index of inequality (RII)		
	2019	2020	2021	2019	2020	2021
BCG (n = 149)	8.3 (−2.7, 19.3)	11.3 (1.0, 21.5)	16.1 (5.5, 26.7)	1.10 (0.96, 1.23)	1.14 (1.01, 1.27)	1.21 (1.06, 1.35)
DTP1 (n = 187)	14.8 (9.8, 19.8)	18.5 (13.9, 23.1)	20.8 (15.2, 26.4)	1.17 (1.11, 1.24)	1.23 (1.16, 1.29)	1.26 (1.18, 1.34)
DTP3 (n = 187)	20.1 (13.7, 26.5)	23.6 (17.5, 29.7)	26.9 (20.0, 33.8)	1.25 (1.16, 1.35)	1.32 (1.22, 1.41)	1.37 (1.25, 1.49)
Pol3 (n = 187)	21.0 (14.7, 27.4)	24.3 (18.3, 30.4)	28.5 (21.3, 35.8)	1.27 (1.17, 1.36)	1.33 (1.23, 1.43)	1.40 (1.27, 1.53)
RCV1 (n = 165)	13.6 (8.3, 18.8)	16.8 (11.4, 22.3)	20.2 (13.8, 26.6)	1.16 (1.09, 1.23)	1.21 (1.13, 1.29)	1.27 (1.17, 1.36)
MCV1 (n = 187)	23.7 (17.1, 30.4)	25.5 (19.1, 32.0)	30.0 (23.0, 37.0)	1.31 (1.21, 1.42)	1.35 (1.24, 1.46)	1.43 (1.30, 1.57)
MCV2 (n = 170)	33.9 (23.9, 43.9)	33.6 (25.1, 42.1)	31.2 (21.5, 40.8)	1.52 (1.31, 1.73)	1.53 (1.35, 1.70)	1.49 (1.29, 1.69)
HepB3 (n = 182)	18.6 (12.0, 25.3)	21.6 (15.3, 28.0)	24.8 (17.6, 32.0)	1.24 (1.14, 1.33)	1.29 (1.19, 1.39)	1.34 (1.22, 1.46)
Hib3 (n = 184)	18.5 (11.3, 25.7)	20.3 (13.2, 27.5)	24.0 (16.4, 31.6)	1.23 (1.13, 1.34)	1.27 (1.16, 1.38)	1.33 (1.20, 1.45)
PCV3 (n = 141)	17.2 (8.9, 25.5)	19.8 (11.8, 27.8)	24.4 (15.8, 33.1)	1.23 (1.10, 1.36)	1.27 (1.14, 1.41)	1.36 (1.20, 1.51)
RotaC (n = 98)	1.3 (–13.4, 15.9)	3.0 (–9.3, 15.3)	7.8 (–3.9, 19.5)	1.02 (0.83, 1.20)	1.04 (0.88, 1.20)	1.11 (0.94, 1.28)

Notes: The analysis encompassed a smaller number of samples, owing to the unavailability of GDP per capita data from the World Bank in eight countries and territories (ISO codes: COK, ERI, NIU, PRK, SSD, SYR, TKM and VEN).

BCG, Bacillus Calmette-Guérin vaccine; DTP, Diphtheria-tetanus-pertussis vaccine; Pol, Polio vaccine; RCV, Rubella-containing vaccine; MCV, Measles-containing vaccine; HepB, Hepatitis B vaccine; Hib, *Haemophilus influenzae* type b vaccine; PCV, Pneumococcal conjugate vaccine, RotaC, Completed rotavirus vaccine.

a SII is measured in percentage points.

**Table 3:** Results of Jonckeere–Terpstra test and Willcoxon-Mann-Whitney rank sum test by region, combing values of 11 vaccines.

Region	p-value of Jonckeere–Terpstra test		p-value of Willcoxon-Mann-Whitney rank sum test	
	SII	RII	SII	RII
Global	0.0319	0.0207	0.0452	0.0355
The African region	0.1809	0.1591	0.3403	0.2912
The region of the Americas	0.0019	0.0006	0.0009	0.0009
The Eastern Mediterranean region	0.5197	0.4671	0.3752	0.2235
The European region	0.1865	0.1800	0.2001	0.1651
The South-East Asian region	0.0000	0.0000	0.0015	0.0003
The Western Pacific region	0.0000	0.0000	0.0002	0.0003

Notes: The Jonckeere–Terpstra test used data in 2019–2021, and the Willcoxon-Mann-Whitney rank sum test used data in 2019 and 2021.

A two-sided p-value below 0.05 was considered statistically significant.

**Table 4:** Global slope index of inequality and relative index of inequality for 11 routine childhood vaccine coverage from 2010 to 2018.

Vaccine type	2010	2011	2012	2013	2014	2015	2016	2017	2018
**Slope index of inequality (SII)**^a^									
BCG	8.9 (1.5, 16.3)	10.2 (2.3, 18.0)	7.2 (0.2, 14.2)	14.4 (5.5, 23.4)	13.6 (5.6, 21.6)	12 (2.7, 21.2)	10.6 (1.1, 20.0)	9 (−1.0, 19.0)	9.2 (−1.7, 20.1)
DTP1	13.9 (9.0, 18.8)	11.3 (6.7, 15.9)	10.8 (6.1, 15.4)	14.3 (8.1, 20.5)	14.6 (9.1, 20.1)	15.4 (10.0, 20.8)	16.3 (11.2, 21.4)	15.6 (10.5, 20.7)	14.3 (9.2, 19.3)
DTP3	21.6 (15.0, 28.2)	18.9 (12.4, 25.4)	17.5 (11.1, 24.0)	22 (14.4, 29.6)	22.5 (15.0, 29.9)	22.4 (15.3, 29.6)	23 (16.4, 29.7)	20.9 (14.2, 27.5)	20 (13.5, 26.5)
Pol3	21.4 (15.2, 27.6)	20.3 (13.6, 27.0)	17.5 (11.4, 23.5)	22.7 (15.3, 30.1)	22.7 (15.7, 29.7)	21.1 (14.6, 27.7)	22 (15.8, 28.1)	20.8 (14.4, 27.2)	21 (14.6, 27.5)
RCV1	4.3 (−1.9, 10.5)	3 (−1.8, 7.7)	6.8 (−0.5, 14.1)	6 (2.0, 10.1)	9.1 (4.0, 14.1)	12.4 (6.6, 18.3)	12.1 (5.7, 18.4)	14.8 (7.1, 22.4)	13.9 (8.4, 19.3)
MCV1	20.9 (14.0, 27.8)	20.2 (14.0, 26.5)	18.6 (12.5, 24.6)	22.1 (14.9, 29.3)	23 (16.0, 30.0)	24.3 (17.6, 31.0)	24.3 (17.5, 31.0)	23.5 (16.8, 30.2)	22.3 (15.5, 29.1)
MCV2	22.4 (8.0, 36.7)	18.6 (5.9, 31.4)	31.2 (17.7, 44.7)	22.9 (13.1, 32.6)	36.9 (24.5, 49.4)	31.8 (20.1, 43.6)	35.2 (24.4, 45.9)	28.6 (19.2, 38.0)	26 (18.0, 34.0)
HepB3	18.7 (11.1, 26.4)	17 (8.5, 25.5)	13.7 (5.6, 21.7)	17.8 (9.3, 26.3)	19.1 (11.0, 27.3)	19.2 (11.4, 27.0)	20 (12.9, 27.1)	17.8 (10.6, 25.0)	18 (11.1, 25.0)
Hib3	22 (14.8, 29.2)	20.9 (14.0, 27.7)	19.5 (13.0, 26.1)	21.5 (12.9, 30.2)	21.8 (13.2, 30.3)	23.5 (15.8, 31.2)	22.4 (15.2, 29.7)	21 (13.9, 28.1)	19.6 (12.6, 26.5)
PCV3	34.9 (3.8, 65.9)	50.3 (25.6, 74.9)	23.6 (4.0, 43.2)	29.7 (14.9, 44.5)	37.3 (23.8, 50.8)	35.2 (23.0, 47.4)	19 (8.5, 29.5)	15.6 (7.9, 23.3)	16.3 (6.3, 26.4)
RotaC	15.2 (−22.1, 52.6)	18.5 (−11.2, 48.2)	38.3 (5.5, 71.1)	28.4 (0.9, 55.8)	26.1 (1.9, 50.3)	15.8 (−2.1, 33.7)	4 (–13.8, 21.7)	−5.9 (−23.3, 11.6)	−1.8 (−17.5, 13.9)
**Relative index of inequality (RII)**									
BCG	1.1 (1.01, 1.19)	1.12 (1.02, 1.21)	1.08 (1.00, 1.16)	1.17 (1.06, 1.29)	1.16 (1.06, 1.26)	1.14 (1.03, 1.26)	1.12 (1.01, 1.24)	1.1 (0.98, 1.22)	1.11 (0.98, 1.24)
DTP1	1.16 (1.10, 1.22)	1.13 (1.07, 1.18)	1.12 (1.07, 1.18)	1.17 (1.09, 1.25)	1.17 (1.10, 1.24)	1.18 (1.11, 1.25)	1.19 (1.12, 1.26)	1.18 (1.12, 1.25)	1.17 (1.10, 1.23)
DTP3	1.28 (1.18, 1.37)	1.24 (1.14, 1.33)	1.22 (1.13, 1.31)	1.28 (1.17, 1.39)	1.29 (1.18, 1.40)	1.29 (1.18, 1.40)	1.3 (1.19, 1.40)	1.27 (1.17, 1.37)	1.25 (1.16, 1.35)
Pol3	1.27 (1.18, 1.37)	1.26 (1.16, 1.35)	1.22 (1.13, 1.30)	1.29 (1.18, 1.40)	1.29 (1.19, 1.40)	1.27 (1.17, 1.37)	1.28 (1.19, 1.38)	1.27 (1.17, 1.36)	1.27 (1.17, 1.37)
RCV1	1.05 (0.98, 1.12)	1.03 (0.98, 1.09)	1.08 (0.99, 1.16)	1.07 (1.02, 1.11)	1.1 (1.04, 1.16)	1.15 (1.07, 1.22)	1.14 (1.06, 1.22)	1.18 (1.07, 1.28)	1.17 (1.09, 1.24)
MCV1	1.27 (1.17, 1.37)	1.26 (1.17, 1.35)	1.23 (1.15, 1.32)	1.28 (1.18, 1.39)	1.3 (1.19, 1.41)	1.32 (1.21, 1.43)	1.32 (1.21, 1.43)	1.31 (1.20, 1.42)	1.29 (1.19, 1.40)
MCV2	1.3 (1.07, 1.53)	1.24 (1.05, 1.43)	1.45 (1.20, 1.70)	1.31 (1.15, 1.47)	1.57 (1.30, 1.83)	1.47 (1.24, 1.69)	1.54 (1.32, 1.76)	1.41 (1.24, 1.59)	1.37 (1.23, 1.51)
HepB3	1.24 (1.13, 1.35)	1.21 (1.09, 1.33)	1.17 (1.06, 1.28)	1.23 (1.10, 1.35)	1.24 (1.13, 1.36)	1.25 (1.13, 1.36)	1.26 (1.15, 1.36)	1.23 (1.12, 1.33)	1.23 (1.13, 1.33)
Hib3	1.29 (1.18, 1.40)	1.27 (1.17, 1.37)	1.25 (1.15, 1.34)	1.28 (1.15, 1.41)	1.28 (1.15, 1.41)	1.31 (1.19, 1.43)	1.29 (1.18, 1.40)	1.27 (1.16, 1.38)	1.25 (1.15, 1.35)
PCV3	1.64 (0.86, 2.41)	2.07 (1.21, 2.92)	1.36 (0.99, 1.72)	1.45 (1.16, 1.75)	1.61 (1.30, 1.93)	1.57 (1.29, 1.84)	1.26 (1.10, 1.42)	1.2 (1.09, 1.32)	1.22 (1.07, 1.37)
RotaC	1.25 (0.55, 1.95)	1.26 (0.77, 1.76)	1.74 (0.79, 2.69)	1.47 (0.88, 2.06)	1.46 (0.92, 2.01)	1.23 (0.94, 1.53)	1.05 (0.81, 1.29)	0.93 (0.72, 1.14)	0.98 (0.78, 1.17)

Notes: BCG, Bacillus Calmette-Guérin vaccine; DTP, diphtheria-tetanus-pertussis vaccine; Pol, Polio vaccine; RCV, rubella-containing vaccine; MCV, measles-containing vaccine; HepB, Hepatitis B vaccine; Hib, *Haemophilus influenzae* type b vaccine; PCV, pneumococcal conjugate vaccine, RotaC, completed rotavirus vaccine.

a SII is measured in percentage points.

The inequalities of routine childhood vaccination coverage within different WHO regions were diverse and varied substantially from 2019 to 2021 (webappendix Table S6). Among six WHO regions, the European Region consistently had the lowest inequalities and the Western Pacific Region had the largest inequalities for many routine childhood vaccination coverage indicators, although inequalities in both regions increased from 2019 to 2021. For instance, the SII of DTP3 coverage in the European Region slightly increased from 3.0 percentage points (−3.0, 9.0) in 2019 to 8.2 percentage points (2.0, 14.3) in 2020, and decreased to 7.4 percentage points (1.2, 13.7) in 2021. However, that in the Western Pacific Region increased from 25.0 percentage points (5.8, 44.1) in 2019 to 26.1 percentage points (4.6, 47.6) in 2020, and further increased to 39.0 percentage points (15.2, 62.8) in 2021. The SII of Pol3 coverage in the European Region was 2.6 percentage points (−3.5, 8.8), 7.6 percentage points (1.7, 13.5), and 6.8 percentage points (0.5, 13.1) respectively in 2019, 2020, and 2021, while that in the Western Pacific Region rose from 23.5 percentage points (6.3, 40.7) in 2019 to 29.1 percentage points (9.5, 48.8) in 2020 and 39.8 percentage points (16.8, 62.9) in 2021. Statistical tests manifested that global increase in coverage inequalities was driven by three of the six regions, including the Region of the Americas, the South-East Asian Region, and the Western Pacific Region (Table 3). Additionally, after adjusting for the multiplicity of comparing 11 vaccines simultaneously using the Bonferroni method, the annual differences remained significant in the three regions (webappendix Table S7).

### Vaccination recovery in 2021 by income groups

**Fig. 3:**
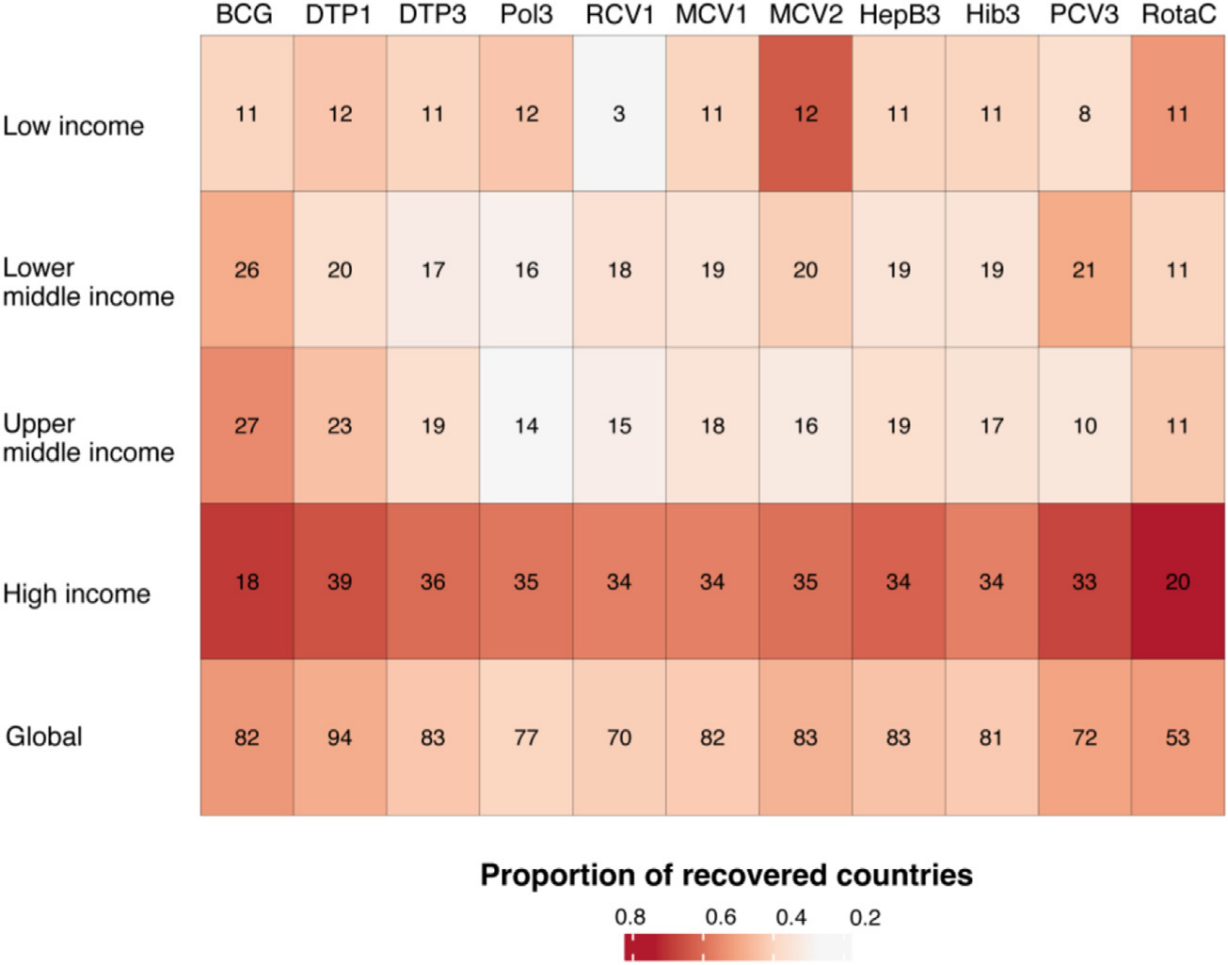
**Number and proportion of countries with vaccine coverage recovering to the 2019 level in 2021 for 11 routine childhood vaccines in four country income groups.** Notes: BCG, Bacillus Calmette-Guérin vaccine; DTP, Diphtheria-tetanus-pertussis vaccine; Pol, Polio vaccine; RCV, Rubella-containing vaccine; MCV, Measles-containing vaccine; HepB, Hepatitis B vaccine; Hib, *Haemophilus influenzae* type b vaccine; PCV, Pneumococcal conjugate vaccine, RotaC, completed rotavirus vaccine.

Compared to 2019, routine childhood vaccination coverage generally decreased at global and regional levels in 2020 and 2021, while we also witnessed some countries with recovered coverage for certain types of vaccines when comparing coverage in 2019 and 2021 (Fig. 3). The recovery status varied substantially by income groups. In 2021, a higher proportion of high-income countries recovered their routine childhood vaccination coverage back to the levels in 2019 compared to low-income and middle-income countries. For example, globally in 2021, 43% of the 195 countries had their DTP3 coverage equivalent to or higher than those in 2019. However, in low, lower-middle, and upper-middle-income countries, the proportions of recovered countries in terms of DTP3 coverage were 39%, 31%, and 36% respectively, all of which were below the global average. In contrast, 60% of high-income countries in 2021 recovered their DTP3 coverage back to the level of 2019 or higher. For 10 of 11 routine childhood vaccination coverage (except MCV2), high-income countries had the highest proportion of recovery in 2021, compared to 2019 before the COVID-19 pandemic.

### Childhood DTP3 vaccination in 2019–2021

**Fig. 4:**
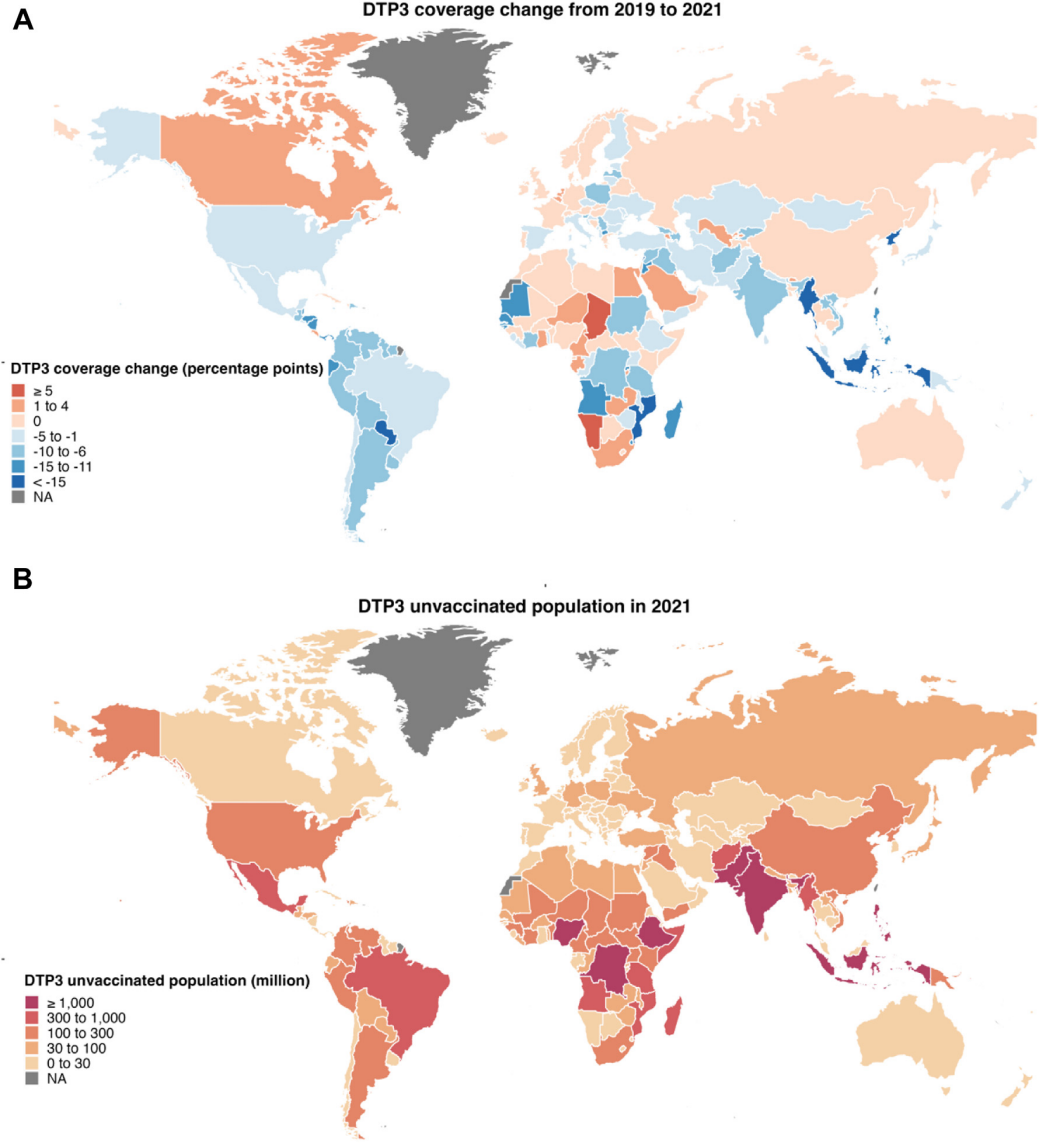
**Global map on DTP3 coverage change from 2019 to 2021 and the number of DTP3-unvaccinated children in 2021.** Note: DTP, Diphtheria-tetanus-pertussis vaccine.

Low-income and lower-middle-income countries had larger declines in DTP3 coverage compared to high and upper-middle-income countries. The top 5 countries with the largest declines in DTP3 coverage from 2019 to 2021 were Pakistan (56%), Myanmar (53%), Vanuatu (28%), Mozambique (27%), and Djibouti (26%), and all of them were low- or lower-middle-income countries (Fig. 4A; webappendix Table S8). In 2021, the number of children unvaccinated with DTP3 was 6.8 million in low-income countries, 14.2 million in lower-middle-income countries, 2.9 million in upper-middle-income countries, and 0.8 million in high-income countries. The top 5 countries with DTP3 unvaccinated children in 2021 were India (3.4 million), Nigeria (3.1 million), Indonesia (1.5 million), Ethiopia (1.3 million), and Democratic Republic of Congo (1.3 million), all from low and lower-middle-income countries, and these top 5 countries accounted for 42.9% of DTP3 unvaccinated children worldwide (Fig. 4B; webappendix Table S9).

## Discussion

During the COVID-19 pandemic, global and regional routine childhood vaccine coverage was severely disrupted in 2020 and further fell in 2021. Routine childhood vaccination coverage in 2021 did not recover to the coverage levels in 2019. The impacts of the COVID-19 pandemic in high-income countries were much smaller than those in low-income countries in terms of GDP per capita. Inequalities of routine childhood vaccine coverage between countries substantially increased from 2019 to 2020, and further rose in 2021 with much larger gaps between high- and low-income countries. Low-income countries with a large population had the largest number of children who did not receive routine vaccination. Among six WHO regions, the impacts of the COVID-19 pandemic were very diverse, with the largest vaccine coverage decline happening in the South-East Asian Region and the lowest occurring in the European Region. Compared to 2019, a huge number of children did not get their routine vaccination in 2020 and 2021, with Pol3 vaccination being most severely disrupted.

DTP and MCV coverage often serve as tracers of inadequate coverage and gaps in the health system.^14^ Causey et al. employed data from 94 countries and territories to estimate disruptions to DTP3 and MCV1 vaccination coverage during the COVID-19 pandemic in 2020.^9^ Shet et al. used data from 170 countries and territories to examine the disruption and recovery of DTP3 and MCV1 due to the COVID-19 pandemic in 2020.^10^ Both studies found significant decreases in DTP3 and MCV1 in 2020 after the COVID-19 pandemic. In addition, both studies found that recovery emerged in the second half of 2020, but need official data to provide updated estimates. The present study reported substantial declines in DTP and MCV coverage in 2020 and 2021 during the COVID-19 pandemic, which might lead to even higher pertussis and measles. Since vaccines against measles were reported to have the largest relative impact on the mortality of children younger than five years in 2000–2019,^2^ the declining MCV1 coverage worldwide requires special attention. For other routine childhood vaccines besides DTP3 and MCV1, the disruption of their coverage during the COVID-19 pandemic was also significant. This study reported global and regional coverage declines in these vaccines from 2019 to 2021, and it was estimated that a large number of children, globally, did not receive major routine childhood vaccines in 2020 and 2021. Such gaps in vaccination coverage may lead to an increasing burden of morbidity and mortality from childhood infectious diseases. Facing the widespread disruption of immunisation services caused by the COVID-19 pandemic,^25^ the results highlighted the importance of vaccination recovery in the near future.

As for global and regional inequalities of routine childhood vaccination coverage and the impacts of the COVID-19 pandemic on these inequalities, the present study found that inequalities persisted at the global and regional levels in 2019 and substantially increased during the COVID-19 pandemic. Previous studies also reported that the COVID-19 pandemic disrupted low-income countries more than high-income countries.^26^, ^27^, ^28^ During the pandemic, wealthier countries tended to have more resources available to invest in maintaining healthcare infrastructure and greater bargaining power; by contrast, low-income countries vulnerable to disruptions in global supply chain may struggle to distribute vaccines effectively to populations, leading to lower vaccination rates and increased inequality.^29^^,^^30^ In this study, significantly increased inequalities of vaccination coverage were observed at the global level with these trends being driven by the Region of the Americas, the South-East Asian Region, and the Western Pacific Region, while other regions did not experience significant increases in coverage inequalities over the period. All six WHO regions had their regional strategic plans for GAVP,^31^ and the regional inequalities of specific routine vaccines in the study could provide evidence for each region to improve its regional strategies.

This study is subject to several limitations. First, the variances in routine childhood vaccine coverage might still exist without the COVID-19 pandemic. Considering the large reductions in routine childhood vaccine coverage in 2020 and 2021, it was reasonable to believe that the entire immunisation service system was severely disrupted and did not recover. Second, there are some concerns about immunisation data quality such as the lack of quantification of uncertainty.^18^ It has been reported that the Americas and Africa are more likely to have potential data quality issues.^32^ Although the quality of data has improved over the past two decades,^32^ data obtained during the COVID-19 pandemic might suffer from further issues. Finally, the study only examined cross-country disparities in routine childhood vaccine coverage, as is typical of prior studies.^33^ The disparity in routine childhood vaccination coverage within a country may also be as great or even greater,^34^^,^^35^ and it is important to assess these within-country inequalities, ideally using individual-level data obtained from population-representative surveys capable of gathering demographic, socioeconomic, and vaccine coverage information across various contexts.^35^

Global and regional inequalities of routine childhood vaccine coverage persisted. The COVID-19 pandemic led global, regional, and national routine childhood coverage of many vaccines to historically low levels in the last decade with immunisation services not having recovered to the 2019 level by 2021. At the same time, inequalities in terms of routine immunisation coverage between low- and high-income countries at the global and regional levels substantially increased. As low-income countries proved less resilient to livelihood and health systems disruptions during the COVID-19 pandemic, vulnerable children in low-income regions and countries could have a higher risk of vaccine-preventable diseases in the coming years, amplifying the burden on health systems. The global community as well as national health systems should prioritise the strengthening of primary health care systems and vaccine programmes in LMICs to protect against adverse outcomes and correct for gaps in routine childhood vaccination coverage. Addressing these global and regional inequalities of routine childhood vaccination will prove critical to the success of IA2030.

## Contributors

XL and HF did the analyses and wrote the first draft of the manuscript. HF designed the project. HZ prepared data. XL, HZ and HF accessed and verified the underlying data. HZ, KBP, BP and MJ provided feedback during the design and interpretation of the project, contributed to data analyses and revisions of the manuscript. HF supervised the entire project. All authors read and approved the final manuscript.

## Data sharing statement

All data were publicly available at official websites of WHO/UNICEF, WB and UN. XL, HZ and HF have directly accessed and verified the underlying data, and take responsibility for the integrity of the data and the accuracy of the data analysis.

## Editorial note

The Lancet Group takes a neutral position with respect to territorial claims in published maps and institutional affiliations.

## Declaration of interests

HF reports grants from BMGF and Sanofi Pasteur. KBP reports grants from EU IMI. BP reports grants from BMGF, Gavi, 10.13039/100004423World Health Organization, USAID and 10.13039/100000001National Science Foundation, and personal fees from Vaxart, Inc, Copenhagen Consensus Center and Costello Medical, Inc. MJ reports grants from NIHR, RCUK, BMGF, Gavi, EU and Wellcome Trust. All other authors declare no competing interests.

## Appendix A Supplementary data

Webappendix Table S1–S9

Abstract in Chinese
