# Supplementary material for: Estimating global and regional between-country inequality in routine childhood vaccine coverage in 195 countries and territories from 2019 to 2021: a longitudinal study
Source: eClinicalMedicine. 2023 Jun 8;60:102042. doi: 10.1016/j.eclinm.2023.102042 (PMC10249397; doi:10.1016/j.eclinm.2023.102042)
Supplement: Webappendix Table S1–S9 [file mmc1.pdf]

# Estimating global and regional between-country inequality in routine childhood vaccine coverage in 195 countries and territories from 2019 to 2021: a longitudinal study

**Authors:** Xiaozhen Lai,[1,2*] Haijun Zhang,[1,3,4*] Koen B. Pouwels,[2] Bryan Patenaude,[3,4] Mark Jit,[5,6,7] Hai Fang,[8,9†]

[1] Department of Health Policy and Management, School of Public Health, Peking University, Beijing, China

[2] Health Economics Research Centre, Nuffield Department of Population Health, University of Oxford, Oxford, UK

[3] Department of International Health, Johns Hopkins Bloomberg School of Public Health, Baltimore, USA

[4] International Vaccine Access Center, Johns Hopkins Bloomberg School of Public Health, Baltimore, USA

[5] Department of Infectious Disease Epidemiology, Faculty of Epidemiology and Population Health, London School of Hygiene and Tropical Medicine, London, UK

[6] Centre for Mathematical Modelling of Infectious Diseases, London School of Hygiene and Tropical Medicine, London, UK

[7] School of Public Health, University of Hong Kong, Hong Kong SAR, China

[8] China Center for Health Development Studies, Peking University, Beijing, China

[9] Peking University Health Science Center-Chinese Center for Disease Control and Prevention Joint Research Center for Vaccine Economics, Peking University, Beijing, China

## Webappendix list

- **Webappendix table 1:** Global coverage rate for 11 routine childhood vaccines from 2010 to 2021
- **Webappendix table 2:** Coverage rate for 11 routine childhood vaccines from 2019 to 2021 by WHO regions
- **Webappendix table 3:** Global slope index of inequality and relative index of inequality for 11 routine childhood vaccine coverage from 2019 to 2021 for all countries and territories with available data
- **Webappendix table 4:** Global slope index of inequality and relative index of inequality for 11 routine childhood vaccine coverage in 2019 using GBD data
- **Webappendix table 5:** Global slope index of inequality and relative index of inequality for 11 routine childhood vaccine coverage from 2019 to 2021 under different grouping strategies
- **Webappendix table 6:** Slope index of inequality and relative index of inequality for 11 routine childhood vaccine coverage from 2019 to 2021 by WHO regions
- **Webappendix table 7:** Bonferroni-adjusted results of Jonckeere-Terpstra test and Willcoxon-Mann-Whitney rank sum test by region, combing values of 11 vaccines
- **Webappendix table 8:** Changes in DTP3 coverage rate from 2019 to 2021 in 195 countries and territories
- **Webappendix table 9:** Number of DTP3 unvaccinated children in 2021 in 195 countries and territories

**Webappendix table 1: Global coverage rate for 11 routine childhood vaccines from 2010 to 2021**

| Vaccine type | 2010 | 2011 | 2012 | 2013 | 2014 | 2015 | 2016 | 2017 | 2018 | 2019 | 2020 | 2021 |
|---|---|---|---|---|---|---|---|---|---|---|---|---|
| BCG | 89.60% (85.4, 93.8) | 89.90% (85.4, 94.4) | 89.80% (84.9, 94.6) | 89.20% (84.2, 94.3) | 88.90% (83.9, 94.0) | 87.90% (82.9, 93.0) | 89.20% (85.0, 93.3) | 89.70% (85.9, 93.6) | 89.80% (86.0, 93.6) | 88.80% (84.8, 92.7) | 85.80% (81.5, 90.2) | 84.40% (80.0, 88.8) |
| DTP1 | 88.90% (84.4, 93.4) | 90.00% (85.7, 94.2) | 89.60% (85.0, 94.2) | 89.30% (84.5, 94.2) | 89.70% (84.8, 94.5) | 89.60% (84.8, 94.4) | 90.20% (86.3, 94.1) | 90.60% (86.9, 94.2) | 90.50% (86.9, 94.1) | 90.50% (86.8, 94.1) | 87.90% (84.4, 91.4) | 86.40% (82.5, 90.3) |
| DTP3 | 83.70% (77.5, 89.8) | 84.70% (79.0, 90.4) | 84.40% (78.4, 90.5) | 84.10% (78.0, 90.2) | 84.80% (78.9, 90.7) | 85.00% (79.0, 91.0) | 86.10% (81.0, 91.2) | 86.10% (81.1, 91.1) | 86.30% (81.2, 91.4) | 86.20% (81.2, 91.3) | 83.20% (78.2, 88.2) | 81.40% (76.1, 86.7) |
| Pol3 | 83.40% (77.0, 89.9) | 83.80% (77.8, 89.8) | 83.90% (77.7, 90.2) | 84.10% (78.0, 90.3) | 84.70% (78.6, 90.7) | 85.30% (79.5, 91.1) | 85.30% (80.1, 90.4) | 85.70% (80.6, 90.8) | 85.90% (80.7, 91.1) | 86.30% (81.4, 91.3) | 82.70% (77.5, 87.9) | 80.90% (75.3, 86.4) |
| RCV1 | 83.20% (69.7, 96.8) | 91.10% (87.5, 94.6) | 89.60% (81.5, 97.7) | 92.80% (90.2, 95.4) | 93.70% (90.7, 96.6) | 92.20% (89.9, 94.4) | 93.30% (90.0, 96.6) | 69.00% (38.0, 100.1) | 89.80% (84.1, 95.4) | 91.50% (88.1, 94.8) | 88.60% (84.9, 92.3) | 86.90% (82.5, 91.4) |
| MCV1 | 84.10% (78.6, 89.7) | 84.50% (78.8, 90.2) | 84.10% (78.1, 90.1) | 84.20% (78.2, 90.2) | 84.60% (78.6, 90.5) | 84.90% (78.8, 90.9) | 85.10% (79.4, 90.8) | 85.50% (80.1, 90.9) | 86.30% (80.7, 91.9) | 86.10% (80.4, 91.9) | 83.70% (78.5, 88.9) | 81.70% (75.9, 87.5) |
| MCV2 | 79.30% (65.8, 92.8) | 67.60% (42.9, 92.2) | 68.20% (48.5, 87.9) | 73.80% (59.2, 88.4) | 73.70% (61.7, 85.8) | 76.10% (66.4, 85.7) | 79.60% (72.3, 86.9) | 81.10% (74.7, 87.6) | 82.70% (76.6, 88.7) | 76.20% (65.9, 86.5) | 76.70% (68.6, 84.8) | 75.80% (67.5, 84.1) |
| HepB3 | 74.70% | 76.30% | 81.90% | 81.10% | 83.30% | 84.30% | 85.50% | 85.50% | 85.60% | 85.60% | 83.00% | 81.20% |

| | | | | | | | | | | | | |
|---|---|---|---|---|---|---|---|---|---|---|---|---|
| | (58.5, 90.8) | (62.0, 90.6) | (74.6, 89.1) | (73.3, 88.8) | (76.9, 89.7) | (78.2, 90.5) | (80.2, 90.7) | (80.3, 90.7) | (80.2, 90.9) | (80.4, 90.9) | (78.0, 88.0) | (75.8, 86.6) |
| Hib3 | 78.40% | 81.10% | 77.80% | 62.00% | 65.60% | 73.60% | 82.10% | 83.70% | 83.80% | 84.00% | 80.70% | 78.90% |
| | (70.5, 86.4) | (74.7, 87.5) | (66.4, 89.2) | (41.8, 82.2) | (45.3, 85.9) | (60.9, 86.3) | (77.9, 86.2) | (79.2, 88.3) | (79.1, 88.5) | (79.2, 88.8) | (76.7, 84.8) | (74.3, 83.6) |
| PCV3 | 70.70% | 65.90% | 73.70% | 73.90% | 74.50% | 72.00% | 75.10% | 78.20% | 59.30% | 61.90% | 61.60% | 60.90% |
| | (51.4, 90.0) | (47.9, 83.9) | (60.9, 86.6) | (65.4, 82.5) | (67.3, 81.7) | (62.9, 81.1) | (68.1, 82.0) | (71.9, 84.5) | (34.9, 83.7) | (40.5, 83.4) | (43.0, 80.3) | (44.3, 77.5) |
| RotaC | 69.50% | 76.20% | 65.30% | 73.40% | 64.40% | 70.40% | 50.60% | 50.10% | 61.10% | 66.80% | 76.20% | 76.50% |
| | (57.0, 82.0) | (64.4, 88.0) | (48.6, 82.1) | (62.8, 84.0) | (52.8, 76.0) | (60.7, 80.1) | (15.8, 85.4) | (24.5, 75.7) | (44.4, 77.7) | (56.1, 77.5) | (70.0, 82.3) | (71.2, 81.8) |

Data source: WHO-UNICEF Estimates of National Immunization Coverage (WUENIC): 2021 revision

Notes: BCG, Bacillus Calmette-Guérin vaccine; DTP, diphtheria-tetanus-pertussis vaccine; Pol, Polio vaccine; RCV, rubella-containing vaccine; MCV, measles-containing vaccine; HepB, Hepatitis B vaccine; Hib, Haemophilus influenzae type b vaccine; PCV, pneumococcal conjugate vaccine, RotaC, completed rotavirus vaccine.

Webappendix table 2: Coverage rate for 11 routine childhood vaccines from 2019 to 2021 by WHO regions

| Vaccine type | The African Region | | | | The Region of the Americas | | | | The Eastern Mediterranean Region | | | |
|---|---|---|---|---|---|---|---|---|---|---|---|---|
| | N | 2019 | 2020 | 2021 | N | 2019 | 2020 | 2021 | N | 2019 | 2020 | 2021 |
| BCG | 47 | 81.6% (76.1, 87.1) | 81.3% (76.8, 85.8) | 78.2% (73.4, 83.1) | 27 | 83.5% (78.8, 88.3) | 68.3% (50.8, 85.9) | 80.6% (67.3, 93.9) | 20 | 88.9% (82.0, 95.8) | 89.4% (83.8, 95.0) | 88.5% (81.8, 95.2) |
| DTP1 | 47 | 82.3% (75.7, 88.9) | 81.1% (75.7, 86.4) | 79.9% (74.2, 85.5) | 35 | 89.1% (82.7, 95.5) | 88.2% (82.1, 94.4) | 86.1% (78.0, 94.1) | 22 | 89.9% (85.1, 94.7) | 86.9% (81.4, 92.5) | 88.8% (83.7, 93.9) |
| DTP3 | 47 | 74.7% (66.2, 83.3) | 73.0% (65.3, 80.8) | 71.3% (63.7, 78.9) | 35 | 83.7% (75.2, 92.2) | 80.7% (72.8, 88.7) | 80.1% (71.0, 89.2) | 22 | 85.5% (79.4, 91.5) | 81.3% (74.1, 88.6) | 82.8% (76.1, 89.4) |
| Pol3 | 47 | 74.3% (65.3, 83.3) | 71.0% (63.0, 79.0) | 69.9% (61.9, 77.9) | 35 | 86.1% (81.3, 90.9) | 79.8% (71.9, 87.7) | 78.8% (69.8, 87.9) | 22 | 85.7% (79.9, 91.6) | 83.9% (77.8, 89.9) | 83.1% (76.8, 89.4) |
| RCV1 | 29 | 81.5% (74.5, 88.5) | 79.0% (71.4, 86.6) | 77.4% (68.8, 86.1) | 35 | 87.3% (82.4, 92.2) | 85.3% (80.5, 90.2) | 84.3% (76.7, 91.8) | 17 | 90.4% (82.8, 98.0) | 90.2% (82.7, 97.7) | 89.6% (81.7, 97.6) |
| MCV1 | 47 | 70.5% (63.1, 78.0) | 69.6% (63.1, 76.1) | 67.9% (60.8, 74.9) | 35 | 87.3% (82.4, 92.2) | 85.3% (80.5, 90.2) | 84.3% (76.7, 91.8) | 22 | 83.6% (76.8, 90.4) | 83.0% (76.7, 89.3) | 81.9% (75.0, 88.8) |
| MCV2 | 32 | 45.7% (23.7, 67.7) | 54.0% (42.5, 65.5) | 54.8% (42.7, 66.8) | 35 | 72.3% (55.7, 88.8) | 72.0% (53.9, 90.1) | 75.1% (57.1, 93.1) | 21 | 78.2% (68.1, 88.3) | 78.9% (69.2, 88.7) | 79.1% (69.4, 88.7) |
| HepB3 | 47 | 74.6% | 72.9% | 71.2% | 35 | 79.3% | 80.7% | 79.6% | 22 | 85.5% | 81.3% | 82.7% |

| | | | | | | | | | | | | |
|---|---|---|---|---|---|---|---|---|---|---|---|---|
| | | (66.1, 83.2) | (65.1, 80.7) | (63.5, 78.8) | | (69.2, 89.5) | (74.0, 87.3) | (71.3, 87.9) | | (79.4, 91.5) | (74.1, 88.6) | (76.1, 89.4) |
| Hib3 | 47 | 74.6% (66.1, 83.2) | 72.8% (65.1, 80.6) | 71.2% (63.5, 78.8) | 35 | 83.1% (76.3, 89.9) | 79.9% (73.1, 86.7) | 79.2% (71.3, 87.2) | 22 | 85.5% (79.5, 91.5) | 81.3% (74.1, 88.6) | 82.8% (76.1, 89.4) |
| PCV3 | 40 | 73.2% (63.3, 83.1) | 71.4% (62.4, 80.4) | 69.3% (60.3, 78.3) | 25 | 83.0% (74.5, 91.6) | 76.8% (70.1, 83.5) | 74.7% (66.9, 82.4) | 16 | 79.6% (69.1, 90.0) | 75.2% (57.9, 92.4) | 74.8% (57.1, 92.5) |
| RotaC | 36 | 69.0% (49.8, 88.2) | 70.6% (57.1, 84.1) | 71.6% (63.3, 79.9) | 21 | 75.9% (68.2, 83.6) | 72.8% (66.5, 79.2) | 71.8% (65.4, 78.2) | 15 | 77.1% (58.8, 95.3) | 79.3% (68.7, 89.9) | 79.3% (67.0, 91.7) |

| Vaccine type | The European Region | | | | The South-East Asian Region | | | | The Western Pacific Region | | | |
|---|---|---|---|---|---|---|---|---|---|---|---|---|
| | **N** | **2019** | **2020** | **2021** | **N** | **2019** | **2020** | **2021** | **N** | **2019** | **2020** | **2021** |
| BCG | 28 | 92.9% (88.7, 97.2) | 93.5% (89.8, 97.2) | 93.0% (89.0, 97.0) | 11 | 92.6% (90.8, 94.5) | 87.2% (82.8, 91.7) | 84.7% (80.5, 89.0) | 24 | 95.2% (87.1, 100.0) | 94.0% (83.7, 100.0) | 90.7% (74.3, 100.0) |
| DTP1 | 53 | 97.5% (96.7, 98.3) | 96.9% (96.0, 97.8) | 96.7% (95.8, 97.6) | 11 | 94.1% (92.4, 95.7) | 88.0% (84.8, 91.3) | 85.8% (79.3, 92.3) | 27 | 95.5% (88.3, 100.0) | 95.2% (87.9, 100.0) | 92.0% (78.9, 100.0) |
| DTP3 | 53 | 95.1% (93.4, 96.9) | 93.8% (91.9, 95.8) | 93.9% (92.1, 95.6) | 11 | 91.1% (88.7, 93.5) | 85.5% (81.7, 89.4) | 82.4% (74.6, 90.3) | 27 | 94.5% (86.1, 100.0) | 94.4% (85.8, 100.0) | 91.3% (77.4, 100.0) |
| Pol3 | 53 | 94.8% (92.9, 96.8) | 94.2% (92.4, 96.0) | 94.1% (92.3, 95.8) | 11 | 90.4% (88.0, 92.8) | 85.1% (81.2, 89.1) | 82.3% (74.5, 90.1) | 27 | 95.3% (88.6, 100.0) | 93.6% (84.1, 100.0) | 91.1% (76.8, 100.0) |

| | | | | | | | | | | | | |
|---|---|---|---|---|---|---|---|---|---|---|---|---|
| RCV1 | 53 | 95.7% (94.4, 97.0) | 93.7% (91.8, 95.7) | 94.1% (92.3, 96.0) | 10 | 93.9% (91.2, 96.7) | 88.2% (83.6, 92.8) | 86.4% (79.1, 93.7) | 27 | 94.9% (87.5, 100.0) | 94.6% (86.4, 100.0) | 91.7% (78.3, 100.0) |
| MCV1 | 53 | 95.7% (94.4, 97.0) | 93.7% (91.8, 95.7) | 94.1% (92.3, 96.0) | 11 | 94.0% (91.3, 96.6) | 88.3% (83.9, 92.7) | 85.9% (78.3, 93.5) | 27 | 95.1% (87.9, 100.0) | 94.6% (86.4, 100.0) | 91.7% (78.3, 100.0) |
| MCV2 | 52 | 92.3% (89.7, 94.8) | 91.6% (89.2, 93.9) | 92.0% (89.7, 94.2) | 11 | 83.1% (78.6, 87.7) | 79.8% (72.7, 86.9) | 77.5% (65.4, 89.5) | 26 | 92.8% (83.5, 100.0) | 93.1% (82.7, 100.0) | 90.1% (74.7, 100.0) |
| HepB3 | 48 | 93.9% (91.5, 96.3) | 92.9% (90.6, 95.2) | 93.0% (90.7, 95.2) | 11 | 91.1% (88.7, 93.5) | 85.5% (81.7, 89.4) | 82.4% (74.6, 90.3) | 27 | 94.6% (86.2, 100.0) | 94.3% (85.5, 100.0) | 91.1% (77.1, 100.0) |
| Hib3 | 52 | 94.5% (92.4, 96.5) | 93.4% (91.5, 95.4) | 93.4% (91.9, 94.9) | 10 | 90.9% (88.5, 93.4) | 85.3% (81.4, 89.2) | 82.1% (73.8, 90.5) | 26 | 83.6% (72.4, 94.8) | 84.2% (72.3, 96.1) | 76.0% (59.8, 92.2) |
| PCV3 | 40 | 89.9% (85.7, 94.1) | 89.1% (85.8, 92.4) | 90.3% (86.8, 93.8) | 6 | 24.2% (-4.4, 52.9) | 28.7% (2.3, 55.2) | 30.1% (6.1, 54.1) | 17 | 70.4% (47.9, 93.0) | 77.0% (61.9, 92.0) | 68.7% (45.6, 91.9) |
| RotaC | 21 | 67.2% (53.4, 81.0) | 78.5% (68.1, 88.9) | 77.5% (69.0, 86.0) | 1 | 53.0% - | 82.0% - | 83.0% - | 8 | 91.1% (88.1, 94.0) | 92.1% (89.4, 94.7) | 93.8% (90.9, 96.8) |

Data source: WHO-UNICEF Estimates of National Immunization Coverage (WUENIC): 2021 revision

Notes: BCG, Bacillus Calmette-Guérin vaccine; DTP, diphtheria-tetanus-pertussis vaccine; Pol, Polio vaccine; RCV, rubella-containing vaccine; MCV, measles-containing vaccine; HepB, Hepatitis B vaccine; Hib, Haemophilus influenzae type b vaccine; PCV, pneumococcal conjugate vaccine, RotaC, completed rotavirus vaccine.

**Webappendix table 3: Global slope index of inequality and relative index of inequality for 11 routine childhood vaccine coverage from 2019 to 2021 for all countries and territories with available data**

| Vaccine type | Slope index of inequality (SII)* | | | Relative index of inequality (RII) | | |
|---|---|---|---|---|---|---|
| | 2019 | 2020 | 2021 | 2019 | 2020 | 2021 |
| BCG (n=149, 149, 149) | 8.3 (-2.7, 19.3) | 11.3 (1.0, 21.5) | 16.1 (5.5, 26.7) | 1.10 (0.96, 1.23) | 1.14 (1.01, 1.27) | 1.21 (1.06, 1.35) |
| DTP1 (n=187, 187, 187) | 14.8 (9.8, 19.8) | 18.5 (13.9, 23.1) | 20.8 (15.2, 26.4) | 1.17 (1.11, 1.24) | 1.23 (1.16, 1.29) | 1.26 (1.18, 1.34) |
| DTP3 (n=187, 187, 187) | 20.1 (13.7, 26.5) | 23.6 (17.5, 29.7) | 26.9 (20.0, 33.8) | 1.25 (1.16, 1.35) | 1.32 (1.22, 1.41) | 1.37 (1.25, 1.49) |
| Pol3 (n=187, 187, 187) | 21.0 (14.7, 27.4) | 24.3 (18.3, 30.4) | 28.5 (21.3, 35.8) | 1.27 (1.17, 1.36) | 1.33 (1.23, 1.43) | 1.40 (1.27, 1.53) |
| RCV1 (n=165, 167, 167) | 13.6 (8.3, 18.8) | 16.6 (11.2, 21.9) | 20.5 (14.1, 27.0) | 1.16 (1.09, 1.23) | 1.21 (1.13, 1.28) | 1.27 (1.17, 1.37) |
| MCV1 (n=187, 187, 187) | 23.7 (17.1, 30.4) | 25.5 (19.1, 32.0) | 30.0 (23.0, 37.0) | 1.31 (1.21, 1.42) | 1.35 (1.24, 1.46) | 1.43 (1.30, 1.57) |
| MCV2 (n=170, 172, 176) | 33.9 (23.9, 43.9) | 36.5 (27.3, 45.7) | 38.9 (28.0, 49.7) | 1.52 (1.31, 1.73) | 1.59 (1.38, 1.80) | 1.67 (1.41, 1.94) |
| HepB3 (n=182, 182, 183) | 18.6 (12.0, 25.3) | 21.6 (15.3, 28.0) | 24.7 (17.5, 31.9) | 1.24 (1.14, 1.33) | 1.29 (1.19, 1.39) | 1.34 (1.22, 1.46) |
| Hib3 (n=184, 185, 185) | 18.5 (11.3, 25.7) | 19.7 (12.5, 27.0) | 23.7 (16.1, 31.2) | 1.23 (1.13, 1.34) | 1.26 (1.15, 1.37) | 1.32 (1.20, 1.45) |
| PCV3 (n=141, 143, 149) | 17.2 (8.9, 25.5) | 20.1 (12.0, 28.1) | 24.8 (15.9, 33.7) | 1.23 (1.10, 1.36) | 1.28 (1.15, 1.41) | 1.37 (1.20, 1.53) |
| RotaC (n=98, 105, 113) | 1.3 (-13.4, 15.9) | 4.8 (-7.1, 16.8) | 6.0 (-5.0, 17.1) | 1.02 (0.83, 1.20) | 1.06 (0.90, 1.23) | 1.08 (0.93, 1.24) |

Data source: WHO-UNICEF Estimates of National Immunization Coverage (WUENIC): 2021 revision

Notes: * SII is measured in percentage points. BCG, Bacillus Calmette-Guérin vaccine; DTP, diphtheria-tetanus-pertussis vaccine; Pol, Polio vaccine; RCV, rubella-containing vaccine; MCV,

measles-containing vaccine; HepB, Hepatitis B vaccine; Hib, Haemophilus influenzae type b vaccine; PCV, pneumococcal conjugate vaccine, RotaC, completed rotavirus vaccine.

**Webappendix table 4: Global slope index of inequality and relative index of inequality for 11 routine childhood vaccine coverage in 2019 using GBD data**

| Vaccine type | Slope index of inequality (SII)* | Relative index of inequality (RII) |
|---|---|---|
| BCG<br>(n=149) | 11.4<br>(5.2, 17.6) | 1.14<br>(1.06, 1.22) |
| DTP1<br>(n=187) | 17.9<br>(15.0, 20.9) | 1.22<br>(1.18, 1.26) |
| DTP3<br>(n=187) | 23.5<br>(19.8, 27.3) | 1.31<br>(1.25, 1.37) |
| Pol3<br>(n=187) | 24.7<br>(20.9, 28.5) | 1.33<br>(1.27, 1.40) |
| RCV1<br>(n=165) | 17.3<br>(14.0, 20.6) | 1.22<br>(1.17, 1.27) |
| MCV1<br>(n=187) | 26.5<br>(22.7, 30.4) | 1.37<br>(1.30, 1.43) |
| MCV2<br>(n=170) | 33.5<br>(28.1, 38.9) | 1.52<br>(1.41, 1.64) |
| HepB3<br>(n=182) | 21.7<br>(17.9, 25.6) | 1.29<br>(1.23, 1.35) |
| Hib3<br>(n=184) | 21.1<br>(16.8, 25.3) | 1.28<br>(1.21, 1.34) |
| PCV3<br>(n=141) | 19.6<br>(14.9, 24.4) | 1.27<br>(1.19, 1.35) |
| RotaC<br>(n=98) | 4.8<br>(-2.5, 12.2) | 1.06<br>(0.96, 1.16) |

Data source: Global Burden of Disease Study 2020, Release 1 (GBD 2020 R1) Routine Childhood Vaccination Coverage 1980-2019

Notes: * SII is measured in percentage points. BCG, Bacillus Calmette-Guérin vaccine; DTP,

diphtheria-tetanus-pertussis vaccine; Pol, Polio vaccine; RCV, rubella-containing vaccine; MCV, measles-containing vaccine; HepB, Hepatitis B vaccine; Hib, Haemophilus influenzae type b vaccine; PCV, pneumococcal conjugate vaccine, RotaC, completed rotavirus vaccine.

**Webappendix table 5: Global slope index of inequality and relative index of inequality for 11 routine childhood vaccine coverage from 2019 to 2021 under different grouping strategies**

| Vaccine type | Slope index of inequality (SII)* | | | Relative index of inequality (RII) | | |
|---|---|---|---|---|---|---|
| | 2019 | 2020 | 2021 | 2019 | 2020 | 2021 |
| **Grouping strategy 1: 4 quartiles** | | | | | | |
| BCG (n=149) | 6.5 (-3.8, 16.8) | 9.6 (-0.7, 19.8) | 15.2 (4.7, 25.6) | 1.08 (0.95, 1.20) | 1.12 (0.99, 1.24) | 1.19 (1.05, 1.34) |
| DTP1 (n=187) | 14.6 (9.6, 19.5) | 18.3 (13.6, 23.0) | 19.6 (13.8, 25.5) | 1.17 (1.11, 1.23) | 1.22 (1.16, 1.29) | 1.24 (1.16, 1.33) |
| DTP3 (n=187) | 20.2 (13.7, 26.6) | 24.3 (18.1, 30.5) | 25 (17.7, 32.3) | 1.26 (1.16, 1.35) | 1.33 (1.23, 1.43) | 1.34 (1.22, 1.46) |
| Pol3 (n=187) | 21.2 (14.8, 27.7) | 25.3 (19.2, 31.4) | 26.6 (18.8, 34.3) | 1.27 (1.18, 1.37) | 1.34 (1.24, 1.45) | 1.37 (1.24, 1.50) |
| RCV1 (n=165) | 14.9 (9.3, 20.5) | 17.4 (11.8, 23.1) | 21.4 (14.7, 28.2) | 1.18 (1.10, 1.25) | 1.22 (1.14, 1.30) | 1.28 (1.18, 1.39) |
| MCV1 (n=187) | 24 (17.4, 30.7) | 26.7 (20.1, 33.3) | 27.4 (19.4, 35.4) | 1.32 (1.21, 1.42) | 1.37 (1.26, 1.49) | 1.39 (1.25, 1.53) |
| MCV2 (n=170) | 33.2 (23.2, 43.2) | 34.9 (26.0, 43.7) | 32.5 (22.9, 42.0) | 1.51 (1.30, 1.71) | 1.55 (1.36, 1.74) | 1.52 (1.32, 1.71) |
| HepB3 (n=182) | 18.9 (12.2, 25.6) | 22.5 (16.1, 28.9) | 22.3 (14.7, 30.0) | 1.24 (1.14, 1.34) | 1.3 (1.20, 1.40) | 1.3 (1.18, 1.43) |
| Hib3 (n=184) | 19.2 (11.2, 27.1) | 21.3 (13.4, 29.1) | 24.5 (16.0, 33.0) | 1.24 (1.13, 1.36) | 1.28 (1.16, 1.40) | 1.34 (1.20, 1.48) |
| PCV3 (n=141) | 17.9 (8.1, 27.6) | 16.6 (6.9, 26.3) | 22.5 (11.9, 33.1) | 1.24 (1.09, 1.39) | 1.23 (1.08, 1.37) | 1.32 (1.14, 1.50) |

| | | | | | | |
|---|---|---|---|---|---|---|
| RotaC (n=98) | 0.7 (-15.5, 16.8) | -0.8 (-14.0, 12.4) | 5.5 (-7.3, 18.3) | 1.01 (0.80, 1.21) | 0.99 (0.82, 1.16) | 1.08 (0.90, 1.25) |
| **Grouping strategy 2: 10 deciles** | | | | | | |
| BCG (n=149) | 9 (-1.8, 19.8) | 11.3 (0.9, 21.7) | 16.1 (4.9, 27.2) | 1.11 (0.97, 1.24) | 1.14 (1.00, 1.27) | 1.2 (1.05, 1.36) |
| DTP1 (n=187) | 14.8 (9.8, 19.7) | 18.3 (13.6, 22.9) | 20 (14.5, 25.5) | 1.17 (1.11, 1.24) | 1.22 (1.16, 1.29) | 1.25 (1.17, 1.33) |
| DTP3 (n=187) | 19.9 (13.6, 26.2) | 23.3 (17.3, 29.4) | 25.9 (19.2, 32.6) | 1.25 (1.16, 1.34) | 1.31 (1.21, 1.41) | 1.36 (1.24, 1.47) |
| Pol3 (n=187) | 21 (14.8, 27.3) | 24.1 (18.1, 30.1) | 27.5 (20.5, 34.5) | 1.27 (1.17, 1.36) | 1.33 (1.23, 1.42) | 1.39 (1.26, 1.51) |
| RCV1 (n=165) | 13.3 (8.1, 18.5) | 16.5 (11.3, 21.6) | 20.3 (14.2, 26.5) | 1.16 (1.09, 1.23) | 1.21 (1.13, 1.28) | 1.27 (1.17, 1.36) |
| MCV1 (n=187) | 23.1 (16.6, 29.7) | 24.9 (18.4, 31.3) | 28.7 (21.9, 35.6) | 1.3 (1.20, 1.41) | 1.34 (1.23, 1.45) | 1.41 (1.29, 1.54) |
| MCV2 (n=170) | 34.5 (24.5, 44.5) | 34.3 (26.0, 42.6) | 31.7 (22.2, 41.1) | 1.53 (1.32, 1.74) | 1.54 (1.36, 1.72) | 1.5 (1.31, 1.69) |
| HepB3 (n=182) | 18 (11.4, 24.6) | 21.2 (14.9, 27.5) | 24 (17.0, 31.0) | 1.23 (1.13, 1.32) | 1.28 (1.18, 1.38) | 1.33 (1.21, 1.45) |
| Hib3 (n=184) | 19.7 (13.3, 26.1) | 21.8 (15.7, 27.9) | 25 (18.3, 31.7) | 1.25 (1.16, 1.34) | 1.29 (1.19, 1.39) | 1.34 (1.23, 1.46) |
| PCV3 (n=141) | 16.6 (8.9, 24.3) | 18.9 (11.7, 26.0) | 23.4 (15.4, 31.4) | 1.22 (1.10, 1.34) | 1.26 (1.15, 1.38) | 1.34 (1.20, 1.48) |
| RotaC (n=98) | 3.5 (-10.3, 17.3) | 4.1 (-7.5, 15.8) | 7.6 (-4.3, 19.5) | 1.05 (0.86, 1.23) | 1.05 (0.90, 1.21) | 1.1 (0.93, 1.28) |
| **Grouping strategy 3: 4 income groups** | | | | | | |
| BCG (n=149) | 9.8 (-0.9, 20.4) | 11.5 (1.3, 21.8) | 17 (6.2, 27.8) | 1.12 (0.98, 1.25) | 1.14 (1.01, 1.27) | 1.22 (1.07, 1.37) |
| DTP1 | 15.8 | 18.2 | 21.7 | 1.19 | 1.22 | 1.28 |

| | | | | | | |
|---|---|---|---|---|---|---|
| (n=187) | (10.7, 20.8) | (13.4, 23.1) | (16.0, 27.5) | (1.12, 1.25) | (1.16, 1.29) | (1.19, 1.36) |
| DTP3 | 21.5 | 23.4 | 27.6 | 1.28 | 1.32 | 1.39 |
| (n=187) | (15.2, 27.7) | (17.2, 29.5) | (20.9, 34.3) | (1.18, 1.37) | (1.22, 1.42) | (1.27, 1.51) |
| Pol3 | 22.1 | 24.6 | 30.3 | 1.29 | 1.34 | 1.44 |
| (n=187) | (16.0, 28.2) | (18.7, 30.5) | (22.4, 38.2) | (1.19, 1.38) | (1.24, 1.44) | (1.29, 1.59) |
| RCV1 | 14.2 | 17.2 | 20.6 | 1.17 | 1.22 | 1.27 |
| (n=165) | (9.1, 19.2) | (11.9, 22.5) | (14.5, 26.8) | (1.10, 1.24) | (1.14, 1.30) | (1.18, 1.37) |
| MCV1 | 23.1 | 25.1 | 29.8 | 1.3 | 1.35 | 1.44 |
| (n=187) | (16.7, 29.4) | (18.9, 31.4) | (23.2, 36.4) | (1.20, 1.41) | (1.24, 1.46) | (1.31, 1.56) |
| MCV2 | 35.3 | 33.8 | 33.1 | 1.55 | 1.54 | 1.54 |
| (n=170) | (25.6, 45.0) | (25.7, 41.9) | (24.2, 42.1) | (1.34, 1.76) | (1.36, 1.71) | (1.34, 1.73) |
| HepB3 | 19.8 | 21.5 | 25.7 | 1.26 | 1.29 | 1.36 |
| (n=182) | (13.3, 26.3) | (15.1, 27.8) | (18.8, 32.6) | (1.16, 1.35) | (1.19, 1.39) | (1.24, 1.48) |
| Hib3 | 21.4 | 23.5 | 27.2 | 1.28 | 1.32 | 1.39 |
| (n=184) | (15.1, 27.8) | (17.3, 29.7) | (20.4, 34.0) | (1.18, 1.38) | (1.22, 1.42) | (1.26, 1.51) |
| PCV3 | 20.7 | 20.3 | 24.1 | 1.29 | 1.29 | 1.35 |
| (n=141) | (11.8, 29.7) | (12.0, 28.6) | (15.2, 33.1) | (1.14, 1.44) | (1.15, 1.42) | (1.19, 1.52) |
| RotaC | 3.1 | 5 | 8.4 | 1.04 | 1.07 | 1.12 |
| (n=98) | (-11.0, 17.3) | (-7.2, 17.1) | (-3.4, 20.3) | (0.85, 1.23) | (0.90, 1.23) | (0.94, 1.29) |

Data source: WHO-UNICEF Estimates of National Immunization Coverage (WUENIC): 2021 revision

Notes: * SII is measured in percentage points. BCG, Bacillus Calmette-Guérin vaccine; DTP, diphtheria-tetanus-pertussis vaccine; Pol, Polio vaccine; RCV, rubella-containing vaccine; MCV, measles-containing vaccine; HepB, Hepatitis B vaccine; Hib, Haemophilus influenzae type b vaccine; PCV, pneumococcal conjugate vaccine, RotaC, completed rotavirus vaccine.

Webappendix table 6: Slope index of inequality and relative index of inequality for 11 routine childhood vaccine coverage from 2019 to 2021 by WHO regions

| Vaccine type | Slope index of inequality (SII)* | | | Relative index of inequality (RII) | | |
|---|---|---|---|---|---|---|
| | 2019 | 2020 | 2021 | 2019 | 2020 | 2021 |
| | | | The African Region | | | |
| BCG (n=45) | 15.7 (4.6, 26.9) | 16.3 (5.7, 26.9) | 23.4 (7.7, 39.1) | 1.20 (1.04, 1.35) | 1.21 (1.06, 1.36) | 1.32 (1.07, 1.58) |
| DTP1 (n=45) | 6.1 (-6.4, 18.7) | 7.1 (-5.7, 19.9) | 10.8 (-4.0, 25.5) | 1.07 (0.92, 1.23) | 1.09 (0.92, 1.25) | 1.14 (0.94, 1.33) |
| DTP3 (n=45) | 8.1 (-9.0, 25.2) | 10.4 (-6.3, 27.1) | 13.8 (-4.8, 32.5) | 1.11 (0.87, 1.34) | 1.14 (0.90, 1.38) | 1.19 (0.91, 1.48) |
| Pol3 (n=45) | 8.9 (-8.1, 25.8) | 10.2 (-6.3, 26.8) | 13.3 (-6.8, 33.5) | 1.12 (0.88, 1.35) | 1.14 (0.90, 1.39) | 1.19 (0.87, 1.51) |
| RCV1 (n=28) | -3.1 (-18.6, 12.4) | -1.6 (-15.0, 11.9) | 0.1 (-14.1, 14.3) | 0.96 (0.79, 1.14) | 0.98 (0.82, 1.14) | 1.00 (0.83, 1.18) |
| MCV1 (n=45) | 8.7 (-10.0, 27.5) | 10.2 (-7.8, 28.1) | 10.7 (-9.9, 31.3) | 1.12 (0.85, 1.39) | 1.15 (0.87, 1.42) | 1.16 (0.83, 1.48) |
| MCV2 (n=31) | 20.1 (-13.8, 53.9) | 17.6 (-6.4, 41.7) | 4.9 (-20.0, 29.8) | 1.37 (0.63, 2.12) | 1.31 (0.81, 1.81) | 1.08 (0.67, 1.49) |
| HepB3 (n=45) | 8.1 (-9.1, 25.3) | 10.6 (-6.2, 27.3) | 13.5 (-5.5, 32.6) | 1.11 (0.87, 1.34) | 1.14 (0.90, 1.39) | 1.19 (0.90, 1.48) |
| Hib3 (n=45) | 8.1 (-9.1, 25.3) | 10.6 (-6.2, 27.3) | 13.5 (-5.5, 32.6) | 1.11 (0.87, 1.34) | 1.14 (0.90, 1.39) | 1.19 (0.90, 1.48) |
| PCV3 (n=39) | 3.9 (-14.9, 22.7) | 12.5 (-3.9, 28.9) | 10.5 (-11.4, 32.4) | 1.05 (0.80, 1.29) | 1.17 (0.92, 1.42) | 1.15 (0.82, 1.47) |
| RotaC (n=35) | 11.0 (-17.5, 39.4) | 6.0 (-17.1, 29.1) | 0.4 (-25.4, 26.2) | 1.15 (0.74, 1.56) | 1.08 (0.76, 1.40) | 1.01 (0.66, 1.35) |
| | | | The Region of the Americas | | | |
| BCG (n=26) | 13.7 (2.8, 24.5) | 14.4 (-0.3, 29.2) | 20.3 (9.5, 31.0) | 1.16 (1.02, 1.30) | 1.18 (0.98, 1.38) | 1.26 (1.10, 1.42) |

| | | | | | | |
|---|---|---|---|---|---|---|
| DTP1 (n=34) | 10.1 (1.8, 18.5) | 17.9 (5.9, 29.8) | 14.9 (5.3, 24.6) | 1.12 (1.01, 1.22) | 1.22 (1.05, 1.39) | 1.19 (1.06, 1.32) |
| DTP3 (n=34) | 11.0 (-1.4, 23.5) | 23.1 (7.4, 38.9) | 17.6 (4.5, 30.7) | 1.13 (0.97, 1.30) | 1.32 (1.06, 1.58) | 1.24 (1.04, 1.44) |
| Pol3 (n=34) | 13.2 (0.3, 26.0) | 24.4 (9.0, 39.9) | 20.9 (7.0, 34.9) | 1.16 (0.99, 1.33) | 1.35 (1.08, 1.61) | 1.30 (1.07, 1.52) |
| RCV1 (n=34) | 11.7 (0.4, 22.9) | 18.2 (4.2, 32.3) | 18.9 (8.0, 29.9) | 1.14 (0.99, 1.29) | 1.24 (1.03, 1.45) | 1.26 (1.09, 1.43) |
| MCV1 (n=34) | 11.7 (0.4, 22.9) | 18.2 (4.2, 32.3) | 18.9 (8.0, 29.9) | 1.14 (0.99, 1.29) | 1.24 (1.03, 1.45) | 1.26 (1.09, 1.43) |
| MCV2 (n=34) | 21.7 (-0.2, 43.6) | 31.8 (8.7, 54.9) | 24.6 (7.0, 42.2) | 1.31 (0.93, 1.69) | 1.52 (1.01, 2.03) | 1.39 (1.05, 1.73) |
| HepB3 (n=34) | 10.4 (-2.5, 23.2) | 21.7 (6.1, 37.3) | 16.7 (3.7, 29.7) | 1.13 (0.96, 1.29) | 1.30 (1.05, 1.55) | 1.23 (1.03, 1.42) |
| Hib3 (n=34) | 12.0 (-0.5, 24.5) | 23.1 (7.3, 39.0) | 17.7 (4.6, 30.9) | 1.15 (0.98, 1.31) | 1.32 (1.06, 1.59) | 1.24 (1.04, 1.44) |
| PCV3 (n=24) | 14.6 (-4.5, 33.8) | 14.2 (-2.0, 30.4) | 21.6 (4.1, 39.1) | 1.19 (0.91, 1.46) | 1.19 (0.95, 1.44) | 1.31 (1.01, 1.62) |
| RotaC (n=20) | -14.9 (-38.4, 8.6) | 12.4 (-7.8, 32.7) | 16.2 (-2.2, 34.6) | 0.83 (0.60, 1.07) | 1.17 (0.87, 1.47) | 1.24 (0.93, 1.55) |
| **The Eastern Mediterranean Region** | | | | | | |
| BCG (n=19) | 18.1 (-20.4, 56.7) | 21.2 (-12.8, 55.2) | 26.7 (-5.0, 58.4) | 1.23 (0.67, 1.79) | 1.28 (0.76, 1.79) | 1.37 (0.83, 1.90) |
| DTP1 (n=21) | 23.3 (-1.8, 48.5) | 16.7 (-7.9, 41.3) | 20.1 (-3.6, 43.7) | 1.29 (0.92, 1.67) | 1.21 (0.86, 1.55) | 1.25 (0.91, 1.60) |
| DTP3 (n=21) | 27.8 (-1.1, 56.7) | 20.0 (-8.7, 48.8) | 31.9 (6.8, 56.9) | 1.37 (0.90, 1.85) | 1.27 (0.82, 1.72) | 1.47 (1.00, 1.94) |
| Pol3 (n=21) | 27.2 (0.1, 54.2) | 16.0 (-11.8, 43.8) | 30.9 (7.4, 54.5) | 1.36 (0.92, 1.81) | 1.21 (0.80, 1.62) | 1.45 (1.02, 1.88) |
| RCV1 | 5.6 | 5.9 | 15.6 | 1.06 | 1.07 | 1.19 |

| | | | | | | |
|---|---|---|---|---|---|---|
| (n=16) | (-21.9, 33.0) | (-20.2, 32.0) | (-11.4, 42.6) | (0.75, 1.38) | (0.76, 1.38) | (0.83, 1.55) |
| MCV1 | 32.5 | 24.9 | 33.2 | 1.46 | 1.35 | 1.50 |
| (n=21) | (5.7, 59.4) | (-1.9, 51.7) | (9.6, 56.8) | (0.97, 1.94) | (0.90, 1.79) | (1.04, 1.95) |
| MCV2 | 41.4 | 38.1 | 47.7 | 1.65 | 1.59 | 1.80 |
| (n=20) | (8.0, 74.7) | (6.4, 69.7) | (23.3, 72.1) | (0.93, 2.37) | (0.92, 2.26) | (1.17, 2.44) |
| HepB3 | 27.1 | 20.2 | 31.3 | 1.37 | 1.27 | 1.46 |
| (n=21) | (-1.9, 56.2) | (-8.6, 48.9) | (6.4, 56.3) | (0.89, 1.84) | (0.82, 1.72) | (0.99, 1.93) |
| Hib3 | 28.1 | 20.0 | 31.9 | 1.38 | 1.27 | 1.47 |
| (n=21) | (-0.7, 56.9) | (-8.7, 48.8) | (6.8, 56.9) | (0.90, 1.86) | (0.82, 1.72) | (1.00, 1.94) |
| PCV3 | 11.4 | -0.6 | 14.4 | 1.14 | 0.99 | 1.20 |
| (n=16) | (-20.8, 43.6) | (-43.1, 41.9) | (-31.2, 59.9) | (0.72, 1.57) | (0.47, 1.51) | (0.53, 1.86) |
| RotaC | -0.3 | -0.1 | 10.6 | 1.00 | 1.00 | 1.14 |
| (n=15) | (-49.7, 49.0) | (-35.9, 35.7) | (-22.4, 43.6) | (0.39, 1.60) | (0.56, 1.44) | (0.67, 1.61) |
| **The European Region** | | | | | | |
| BCG | -27.1 | -27.2 | -30.3 | 0.74 | 0.73 | 0.71 |
| (n=27) | (-67.1, 12.8) | (-66.7, 12.2) | (-69.4, 8.9) | (0.38, 1.09) | (0.38, 1.09) | (0.36, 1.05) |
| DTP1 | 1.5 | 5.0 | 5.0 | 1.02 | 1.05 | 1.05 |
| (n=52) | (-1.7, 4.6) | (1.6, 8.5) | (1.9, 8.1) | (0.98, 1.05) | (1.02, 1.09) | (1.02, 1.09) |
| DTP3 | 3.0 | 8.2 | 7.4 | 1.03 | 1.09 | 1.08 |
| (n=52) | (-3.0, 9.0) | (2.0, 14.3) | (1.2, 13.7) | (0.97, 1.10) | (1.02, 1.17) | (1.01, 1.16) |
| Pol3 | 2.6 | 7.6 | 6.8 | 1.03 | 1.09 | 1.08 |
| (n=52) | (-3.5, 8.8) | (1.7, 13.5) | (0.5, 13.1) | (0.96, 1.10) | (1.02, 1.15) | (1.00, 1.15) |
| RCV1 | 7.2 | 16.2 | 12.1 | 1.08 | 1.20 | 1.14 |
| (n=52) | (-5.4, 19.8) | (2.6, 29.8) | (1.8, 22.3) | (0.93, 1.23) | (1.01, 1.39) | (1.01, 1.28) |
| MCV1 | 7.2 | 16.2 | 12.1 | 1.08 | 1.20 | 1.14 |
| (n=52) | (-5.4, 19.8) | (2.6, 29.8) | (1.8, 22.3) | (0.93, 1.23) | (1.01, 1.39) | (1.01, 1.28) |
| MCV2 | -5.5 | 3.9 | -6.7 | 0.94 | 1.05 | 0.93 |
| (n=51) | (-11.5, 0.6) | (-4.0, 11.8) | (-22.9, 9.5) | (0.88, 1.00) | (0.95, 1.14) | (0.75, 1.10) |
| HepB3 | 1.3 | 8.8 | 6.1 | 1.01 | 1.10 | 1.07 |
| (n=47) | (-8.4, 11.0) | (-1.5, 19.2) | (-2.4, 14.7) | (0.91, 1.12) | (0.98, 1.23) | (0.97, 1.17) |

| | | | | | | |
|---|---|---|---|---|---|---|
| Hib3 (n=51) | 3.6 (-2.8, 10.1) | 8.6 (2.1, 15.2) | 7.0 (0.8, 13.3) | 1.04 (0.97, 1.11) | 1.10 (1.02, 1.18) | 1.08 (1.01, 1.15) |
| PCV3 (n=40) | 0.0 (-7.6, 7.6) | 6.0 (-1.4, 13.4) | 3.4 (-3.6, 10.4) | 1.00 (0.92, 1.09) | 1.07 (0.98, 1.16) | 1.04 (0.96, 1.12) |
| RotaC (n=20) | 27.1 (-9.9, 64.1) | 14.4 (-21.2, 49.9) | 18.8 (-16.1, 53.6) | 1.46 (0.64, 2.28) | 1.21 (0.61, 1.82) | 1.29 (0.65, 1.93) |
| **The South-East Asian Region** | | | | | | |
| BCG (n=10) | 4.2 (-6.6, 15.1) | 16.2 (3.1, 29.4) | 32.3 (-16.9, 81.4) | 1.05 (0.93, 1.16) | 1.19 (1.02, 1.36) | 1.44 (0.57, 2.31) |
| DTP1 (n=10) | 7.6 (-2.6, 17.8) | 13.5 (-1.0, 28.1) | 28.8 (-24.7, 82.4) | 1.08 (0.97, 1.20) | 1.16 (0.98, 1.34) | 1.39 (0.48, 2.31) |
| DTP3 (n=10) | 8.0 (-4.7, 20.6) | 15.3 (-4.0, 34.6) | 30.8 (-31.5, 93.1) | 1.09 (0.94, 1.23) | 1.19 (0.94, 1.43) | 1.44 (0.30, 2.58) |
| Pol3 (n=10) | 9.4 (-3.4, 22.1) | 14.3 (-6.0, 34.7) | 28.7 (-27.5, 84.9) | 1.11 (0.96, 1.25) | 1.17 (0.92, 1.43) | 1.40 (0.43, 2.38) |
| RCV1 (n=10) | 12.0 (-1.8, 25.8) | 9.4 (-11.2, 30.0) | 29.3 (-18.9, 77.6) | 1.14 (0.97, 1.31) | 1.11 (0.86, 1.36) | 1.42 (0.53, 2.31) |
| MCV1 (n=10) | 13.2 (-2.0, 28.4) | 10.3 (-12.4, 33.1) | 32.3 (-20.8, 85.4) | 1.15 (0.96, 1.34) | 1.12 (0.84, 1.40) | 1.46 (0.48, 2.44) |
| MCV2 (n=10) | 17.3 (-7.2, 41.8) | 10.1 (-25.2, 45.4) | 22.7 (-38.4, 83.7) | 1.22 (0.88, 1.56) | 1.13 (0.67, 1.59) | 1.33 (0.30, 2.36) |
| HepB3 (n=10) | 8.0 (-4.7, 20.6) | 15.7 (-3.7, 35.2) | 30.8 (-31.5, 93.1) | 1.09 (0.94, 1.23) | 1.19 (0.94, 1.44) | 1.44 (0.30, 2.58) |
| Hib3 (n=9) | 7.5 (-8.2, 23.2) | 14.5 (-9.4, 38.4) | 29.0 (-39.5, 97.5) | 1.08 (0.90, 1.26) | 1.18 (0.87, 1.48) | 1.42 (0.18, 2.66) |
| PCV3 (n=6) | -69.8 (-216.4, 76.8) | -17.2 (-219.1, 184.7) | 7.2 (-202.0, 216.4) | 0.25 (-0.60, 1.11) | 0.77 (-1.67, 3.20) | 1.14 (-3.05, 5.32) |
| RotaC (n=1) | - | - | - | - | - | - |

| | | | | | | |
|---|---|---|---|---|---|---|
| **The Western Pacific Region** | | | | | | |
| BCG | 25.6 | 28.3 | 42.6 | 1.32 | 1.36 | 1.64 |
| (n=22) | (8.0, 43.2) | (7.7, 48.8) | (15.7, 69.6) | (1.05, 1.60) | (1.03, 1.69) | (1.07, 2.21) |
| DTP1 | 13.5 | 20.6 | 29.4 | 1.15 | 1.25 | 1.38 |
| (n=25) | (-3.0, 29.9) | (0.7, 40.5) | (5.2, 53.6) | (0.94, 1.36) | (0.97, 1.52) | (0.99, 1.77) |
| DTP3 | 25.0 | 26.1 | 39.0 | 1.32 | 1.34 | 1.57 |
| (n=25) | (5.8, 44.1) | (4.6, 47.6) | (15.2, 62.8) | (1.01, 1.63) | (1.00, 1.68) | (1.09, 2.06) |
| Pol3 | 23.5 | 29.1 | 39.8 | 1.30 | 1.39 | 1.60 |
| (n=25) | (6.3, 40.7) | (9.5, 48.8) | (16.8, 62.9) | (1.03, 1.57) | (1.06, 1.72) | (1.11, 2.08) |
| RCV1 | 21.3 | 29.2 | 47.1 | 1.27 | 1.40 | 1.77 |
| (n=25) | (3.7, 38.8) | (12.6, 45.8) | (26.8, 67.5) | (1.00, 1.54) | (1.11, 1.68) | (1.27, 2.27) |
| MCV1 | 20.9 | 29.2 | 47.1 | 1.26 | 1.40 | 1.77 |
| (n=25) | (3.2, 38.5) | (12.6, 45.8) | (26.8, 67.5) | (1.00, 1.53) | (1.11, 1.68) | (1.27, 2.27) |
| MCV2 | 36.8 | 44.7 | 62.0 | 1.58 | 1.76 | 2.36 |
| (n=24) | (8.6, 65.0) | (19.0, 70.5) | (35.7, 88.3) | (0.94, 2.22) | (1.10, 2.43) | (1.28, 3.44) |
| HepB3 | 24.4 | 24.9 | 36.7 | 1.31 | 1.32 | 1.53 |
| (n=25) | (5.5, 43.3) | (3.2, 46.6) | (12.5, 61.0) | (1.01, 1.62) | (0.98, 1.66) | (1.06, 2.00) |
| Hib3 | 19.9 | 24.6 | 41.5 | 1.26 | 1.32 | 1.64 |
| (n=24) | (-6.8, 46.5) | (0.4, 48.7) | (13.4, 69.6) | (0.86, 1.65) | (0.94, 1.70) | (1.03, 2.25) |
| PCV3 | 19.4 | 20.1 | 32.9 | 1.28 | 1.28 | 1.51 |
| (n=16) | (-20.6, 59.3) | (-14.1, 54.3) | (-4.3, 70.0) | (0.60, 1.95) | (0.72, 1.84) | (0.74, 2.28) |
| RotaC | 30.5 | 39.2 | 47.2 | 1.47 | 1.67 | 1.88 |
| (n=7) | (-35.3, 96.3) | (-19.1, 97.6) | (-10.1, 104.5) | (0.05, 2.89) | (0.09, 3.25) | (-0.01, 3.77) |

Data source: WHO-UNICEF Estimates of National Immunization Coverage (WUENIC): 2021 revision

Notes: * SII is measured in percentage points. BCG, Bacillus Calmette-Guérin vaccine; DTP, diphtheria-tetanus-pertussis vaccine; Pol, Polio vaccine; RCV1, rubella-containing vaccine; MCV, measles-containing vaccine; HepB, Hepatitis B vaccine; Hib, Haemophilus influenzae type b vaccine; PCV, pneumococcal conjugate vaccine, RotaC, completed rotavirus vaccine.

**Webappendix table 7: Bonferroni-adjusted results of Jonckeere-Terpstra test and Willcoxon-Mann-Whitney rank sum test by region, combing values of 11 vaccines**

| Region | *P*-value of Jonckeere-Terpstra test | | *P*-value of Willcoxon-Mann-Whitney rank sum test | |
|---|---|---|---|---|
| | SII | RII | SII | RII |
| Global | 0.3506 | 0.2272 | 0.4972 | 0.3906 |
| The African Region | 1.0000 | 1.0000 | 1.0000 | 1.0000 |
| The Region of the Americas | 0.0210 | 0.0063 | 0.0100 | 0.0096 |
| The Eastern Mediterranean Region | 1.0000 | 1.0000 | 1.0000 | 1.0000 |
| The European Region | 1.0000 | 1.0000 | 1.0000 | 1.0000 |
| The South-East Asian Region | 0.0004 | 0.0001 | 0.0163 | 0.0035 |
| The Western Pacific Region | 0.0001 | 0.0003 | 0.0023 | 0.0029 |

Data source: WHO-UNICEF Estimates of National Immunization Coverage (WUENIC): 2021 revision

Notes: The Jonckeere-Terpstra test used data in 2019-2021, and the Willcoxon-Mann-Whitney rank sum test used data in 2019 and 2021. Since the commands do not directly incorporate multiplicity correction methods, the Bonferroni adjustment was performed by taking the observed (uncorrected) p-values and multiplying them by the number of comparisons (11 vaccines). P-values larger than 1.0 after adjustment were recorded as 1.0. A two-sided *p*-value below 0.05 was considered statistically significant.

**Webappendix table 8: Changes in DTP3 coverage rate from 2019 to 2021 in 195 countries and territories**

| Income group | Country code | Country name | DTP3 coverage 2019 (%) | 2021 (%) | Difference (Percentage points) |
|---|---|---|---|---|---|
| Low | AFG | Afghanistan | 72 | 66 | -6 |
| Low | BDI | Burundi | 93 | 94 | 1 |
| Low | BFA | Burkina Faso | 91 | 91 | 0 |
| Low | CAF | Central African Republic | 42 | 42 | 0 |
| Low | COD | Congo, Dem. Rep. | 73 | 65 | -8 |
| Low | ERI | Eritrea | 95 | 95 | 0 |
| Low | ETH | Ethiopia | 68 | 65 | -3 |
| Low | GIN | Guinea | 47 | 47 | 0 |
| Low | GMB | Gambia, The | 88 | 82 | -6 |
| Low | GNB | Guinea-Bissau | 78 | 67 | -11 |
| Low | LBR | Liberia | 70 | 66 | -4 |
| Low | MDG | Madagascar | 68 | 55 | -13 |
| Low | MLI | Mali | 77 | 77 | 0 |
| Low | MOZ | Mozambique | 88 | 61 | -27 |
| Low | MWI | Malawi | 95 | 93 | -2 |
| Low | NER | Niger | 81 | 82 | 1 |
| Low | PRK | Korea, Dem. People's Rep. | 97 | 41 | -56 |
| Low | RWA | Rwanda | 98 | 88 | -10 |
| Low | SDN | Sudan | 93 | 84 | -9 |
| Low | SLE | Sierra Leone | 95 | 92 | -3 |
| Low | SOM | Somalia | 42 | 42 | 0 |
| Low | SSD | South Sudan | 49 | 49 | 0 |
| Low | SYR | Syrian Arab Republic | 54 | 48 | -6 |
| Low | TCD | Chad | 50 | 58 | 8 |
| Low | TGO | Togo | 84 | 83 | -1 |
| Low | UGA | Uganda | 93 | 91 | -2 |
| Low | YEM | Yemen, Rep. | 73 | 72 | -1 |
| Low | ZMB | Zambia | 88 | 91 | 3 |
| Lower middle | AGO | Angola | 57 | 45 | -12 |
| Lower middle | BEN | Benin | 76 | 76 | 0 |
| Lower middle | BGD | Bangladesh | 98 | 98 | 0 |
| Lower middle | BOL | Bolivia | 75 | 70 | -5 |
| Lower middle | BTN | Bhutan | 97 | 98 | 1 |
| Lower middle | CIV | Côte d'Ivoire | 81 | 76 | -5 |
| Lower middle | CMR | Cameroon | 67 | 69 | 2 |
| Lower middle | COG | Congo, Rep. | 79 | 77 | -2 |
| Lower middle | COM | Comoros | 91 | 85 | -6 |
| Lower middle | CPV | Cabo Verde | 96 | 93 | -3 |

| | | | | | |
|---|---|---|---|---|---|
| Lower middle | DJI | Djibouti | 85 | 59 | -26 |
| Lower middle | DZA | Algeria | 91 | 91 | 0 |
| Lower middle | EGY | Egypt, Arab Rep. | 95 | 96 | 1 |
| Lower middle | FSM | Micronesia, Fed. Sts. | 78 | 72 | -6 |
| Lower middle | GHA | Ghana | 97 | 98 | 1 |
| Lower middle | HND | Honduras | 88 | 77 | -11 |
| Lower middle | HTI | Haiti | 51 | 51 | 0 |
| Lower middle | IDN | Indonesia | 85 | 67 | -18 |
| Lower middle | IND | India | 91 | 85 | -6 |
| Lower middle | IRN | Iran, Islamic Rep. | 99 | 98 | -1 |
| Lower middle | KEN | Kenya | 91 | 91 | 0 |
| Lower middle | KGZ | Kyrgyz Republic | 95 | 89 | -6 |
| Lower middle | KHM | Cambodia | 92 | 92 | 0 |
| Lower middle | KIR | Kiribati | 97 | 92 | -5 |
| Lower middle | LAO | Lao PDR | 80 | 75 | -5 |
| Lower middle | LBN | Lebanon | 83 | 67 | -16 |
| Lower middle | LKA | Sri Lanka | 99 | 96 | -3 |
| Lower middle | LSO | Lesotho | 87 | 87 | 0 |
| Lower middle | MAR | Morocco | 99 | 99 | 0 |
| Lower middle | MMR | Myanmar | 90 | 37 | -53 |
| Lower middle | MNG | Mongolia | 98 | 95 | -3 |
| Lower middle | MRT | Mauritania | 80 | 68 | -12 |
| Lower middle | NGA | Nigeria | 56 | 56 | 0 |
| Lower middle | NIC | Nicaragua | 98 | 87 | -11 |
| Lower middle | NPL | Nepal | 93 | 91 | -2 |
| Lower middle | PAK | Pakistan | 84 | 83 | -1 |
| Lower middle | PHL | Philippines | 71 | 57 | -14 |
| Lower middle | PNG | Papua New Guinea | 35 | 31 | -4 |
| Lower middle | PSE | West Bank and Gaza | 99 | 95 | -4 |
| Lower middle | SEN | Senegal | 95 | 85 | -10 |
| Lower middle | SLB | Solomon Islands | 94 | 87 | -7 |
| Lower middle | SLV | El Salvador | 81 | 79 | -2 |
| Lower middle | STP | São Tomé and Príncipe | 95 | 97 | 2 |
| Lower middle | SWZ | Eswatini | 90 | 77 | -13 |
| Lower middle | TJK | Tajikistan | 97 | 97 | 0 |
| Lower middle | TLS | Timor-Leste | 90 | 86 | -4 |
| Lower middle | TUN | Tunisia | 98 | 97 | -1 |
| Lower middle | TZA | Tanzania | 89 | 81 | -8 |
| Lower middle | UKR | Ukraine | 80 | 78 | -2 |
| Lower middle | UZB | Uzbekistan | 96 | 98 | 2 |
| Lower middle | VEN | Venezuela, RB | 64 | 56 | -8 |
| Lower middle | VNM | Vietnam | 89 | 83 | -6 |
| Lower middle | VUT | Vanuatu | 90 | 62 | -28 |
| Lower middle | WSM | Samoa | 68 | 85 | 17 |

| | | | | | |
|---|---|---|---|---|---|
| Lower middle | ZWE | Zimbabwe | 90 | 86 | -4 |
| Upper middle | ALB | Albania | 99 | 98 | -1 |
| Upper middle | ARG | Argentina | 83 | 76 | -7 |
| Upper middle | ARM | Armenia | 92 | 93 | 1 |
| Upper middle | AZE | Azerbaijan | 94 | 89 | -5 |
| Upper middle | BGR | Bulgaria | 93 | 89 | -4 |
| Upper middle | BIH | Bosnia and Herzegovina | 73 | 73 | 0 |
| Upper middle | BLR | Belarus | 98 | 98 | 0 |
| Upper middle | BLZ | Belize | 98 | 83 | -15 |
| Upper middle | BRA | Brazil | 70 | 68 | -2 |
| Upper middle | BWA | Botswana | 95 | 95 | 0 |
| Upper middle | CHN | China | 99 | 99 | 0 |
| Upper middle | COL | Colombia | 94 | 86 | -8 |
| Upper middle | CRI | Costa Rica | 95 | 99 | 4 |
| Upper middle | CUB | Cuba | 99 | 99 | 0 |
| Upper middle | DMA | Dominica | 99 | 92 | -7 |
| Upper middle | DOM | Dominican Republic | 89 | 84 | -5 |
| Upper middle | ECU | Ecuador | 85 | 72 | -13 |
| Upper middle | FJI | Fiji | 99 | 99 | 0 |
| Upper middle | GAB | Gabon | 70 | 75 | 5 |
| Upper middle | GEO | Georgia | 94 | 85 | -9 |
| Upper middle | GNQ | Equatorial Guinea | 53 | 53 | 0 |
| Upper middle | GRD | Grenada | 92 | 72 | -20 |
| Upper middle | GTM | Guatemala | 85 | 79 | -6 |
| Upper middle | GUY | Guyana | 99 | 91 | -8 |
| Upper middle | IRQ | Iraq | 84 | 78 | -6 |
| Upper middle | JAM | Jamaica | 96 | 90 | -6 |
| Upper middle | JOR | Jordan | 89 | 77 | -12 |
| Upper middle | KAZ | Kazakhstan | 97 | 95 | -2 |
| Upper middle | LBY | Libya | 73 | 73 | 0 |
| Upper middle | LCA | St. Lucia | 92 | 80 | -12 |
| Upper middle | MDA | Moldova | 91 | 87 | -4 |
| Upper middle | MDV | Maldives | 98 | 96 | -2 |
| Upper middle | MEX | Mexico | 82 | 78 | -4 |
| Upper middle | MHL | Marshall Islands | 79 | 86 | 7 |
| Upper middle | MKD | North Macedonia | 92 | 81 | -11 |
| Upper middle | MNE | Montenegro | 85 | 83 | -2 |
| Upper middle | MUS | Mauritius | 96 | 92 | -4 |
| Upper middle | MYS | Malaysia | 98 | 95 | -3 |
| Upper middle | NAM | Namibia | 87 | 93 | 6 |
| Upper middle | PER | Peru | 88 | 82 | -6 |
| Upper middle | PLW | Palau | 97 | 95 | -2 |
| Upper middle | PRY | Paraguay | 86 | 70 | -16 |
| Upper middle | RUS | Russian Federation | 97 | 97 | 0 |

| | | | | | |
|---|---|---|---|---|---|
| Upper middle | SRB | Serbia | 97 | 92 | -5 |
| Upper middle | SUR | Suriname | 77 | 72 | -5 |
| Upper middle | THA | Thailand | 97 | 97 | 0 |
| Upper middle | TKM | Turkmenistan | 99 | 97 | -2 |
| Upper middle | TON | Tonga | 99 | 99 | 0 |
| Upper middle | TUR | Türkiye | 99 | 95 | -4 |
| Upper middle | TUV | Tuvalu | 92 | 94 | 2 |
| Upper middle | VCT | St. Vincent and the Grenadines | 97 | 97 | 0 |
| Upper middle | ZAF | South Africa | 85 | 86 | 1 |
| High | AND | Andorra | 99 | 99 | 0 |
| High | ARE | United Arab Emirates | 99 | 96 | -3 |
| High | ATG | Antigua and Barbuda | 95 | 92 | -3 |
| High | AUS | Australia | 95 | 95 | 0 |
| High | AUT | Austria | 85 | 85 | 0 |
| High | BEL | Belgium | 97 | 98 | 1 |
| High | BHR | Bahrain | 99 | 98 | -1 |
| High | BHS | Bahamas, The | 89 | 75 | -14 |
| High | BRB | Barbados | 90 | 82 | -8 |
| High | BRN | Brunei Darussalam | 99 | 99 | 0 |
| High | CAN | Canada | 91 | 92 | 1 |
| High | CHE | Switzerland | 96 | 96 | 0 |
| High | CHL | Chile | 96 | 95 | -1 |
| High | COK | Cook Islands | 98 | 98 | 0 |
| High | CYP | Cyprus | 96 | 96 | 0 |
| High | CZE | Czech Republic | 97 | 94 | -3 |
| High | DEU | Germany | 91 | 91 | 0 |
| High | DNK | Denmark | 97 | 97 | 0 |
| High | ESP | Spain | 95 | 92 | -3 |
| High | EST | Estonia | 91 | 90 | -1 |
| High | FIN | Finland | 91 | 89 | -2 |
| High | FRA | France | 96 | 96 | 0 |
| High | GBR | United Kingdom | 93 | 93 | 0 |
| High | GRC | Greece | 99 | 99 | 0 |
| High | HRV | Croatia | 94 | 92 | -2 |
| High | HUN | Hungary | 99 | 99 | 0 |
| High | IRL | Ireland | 94 | 94 | 0 |
| High | ISL | Iceland | 92 | 92 | 0 |
| High | ISR | Israel | 98 | 98 | 0 |
| High | ITA | Italy | 96 | 94 | -2 |
| High | JPN | Japan | 98 | 96 | -2 |
| High | KNA | St. Kitts and Nevis | 97 | 96 | -1 |
| High | KOR | Korea, Rep. | 98 | 98 | 0 |
| High | KWT | Kuwait | 92 | 94 | 2 |

| | | | | | |
|---|---|---|---|---|---|
| High | LTU | Lithuania | 92 | 90 | -2 |
| High | LUX | Luxembourg | 99 | 99 | 0 |
| High | LVA | Latvia | 99 | 94 | -5 |
| High | MCO | Monaco | 99 | 99 | 0 |
| High | MLT | Malta | 98 | 99 | 1 |
| High | NIU | Niue | 99 | 99 | 0 |
| High | NLD | Netherlands | 94 | 95 | 1 |
| High | NOR | Norway | 97 | 97 | 0 |
| High | NRU | Nauru | 96 | 98 | 2 |
| High | NZL | New Zealand | 92 | 90 | -2 |
| High | OMN | Oman | 99 | 99 | 0 |
| High | PAN | Panama | 88 | 74 | -14 |
| High | POL | Poland | 95 | 90 | -5 |
| High | PRT | Portugal | 99 | 99 | 0 |
| High | QAT | Qatar | 98 | 98 | 0 |
| High | ROU | Romania | 88 | 86 | -2 |
| High | SAU | Saudi Arabia | 96 | 97 | 1 |
| High | SGP | Singapore | 96 | 96 | 0 |
| High | SMR | San Marino | 88 | 90 | 2 |
| High | SVK | Slovak Republic | 97 | 97 | 0 |
| High | SVN | Slovenia | 95 | 86 | -9 |
| High | SWE | Sweden | 98 | 98 | 0 |
| High | SYC | Seychelles | 99 | 94 | -5 |
| High | TTO | Trinidad and Tobago | 93 | 94 | 1 |
| High | URY | Uruguay | 94 | 89 | -5 |
| High | USA | United States | 94 | 93 | -1 |
| | | World weighted average | 86.2 | 81.4 | -4.8 |

Data source: WHO-UNICEF Estimates of National Immunization Coverage (WUENIC): 2021 revision

**Webappendix table 9: Number of DTP3 unvaccinated children in 2021 in 195 countries and territories**

| Income group | Country code | Country name | Children <1 year in 2021 (thousand) | DTP3 coverage in 2021 | Unvaccinated children in 2021 (thousand) |
|---|---|---|---|---|---|
| Low | AFG | Afghanistan | 1324 | 66% | 450 |
| Low | BDI | Burundi | 415 | 94% | 25 |
| Low | BFA | Burkina Faso | 728 | 91% | 66 |
| Low | CAF | Central African Republic | 204 | 42% | 118 |
| Low | COD | Congo, Dem. Rep. | 3657 | 65% | 1280 |
| Low | ERI | Eritrea | 98 | 95% | 5 |
| Low | ETH | Ethiopia | 3669 | 65% | 1284 |
| Low | GIN | Guinea | 426 | 47% | 226 |
| Low | GMB | Gambia, The | 83 | 82% | 15 |
| Low | GNB | Guinea-Bissau | 60 | 67% | 20 |
| Low | LBR | Liberia | 151 | 66% | 51 |
| Low | MDG | Madagascar | 845 | 55% | 380 |
| Low | MLI | Mali | 825 | 77% | 190 |
| Low | MOZ | Mozambique | 1077 | 61% | 420 |
| Low | MWI | Malawi | 619 | 93% | 43 |
| Low | NER | Niger | 1037 | 82% | 187 |
| Low | PRK | Korea, Dem. People's Rep. | 341 | 41% | 201 |
| Low | RWA | Rwanda | 384 | 88% | 46 |
| Low | SDN | Sudan | 1449 | 84% | 232 |
| Low | SLE | Sierra Leone | 241 | 92% | 19 |
| Low | SOM | Somalia | 662 | 42% | 384 |
| Low | SSD | South Sudan | 285 | 49% | 145 |
| Low | SYR | Syrian Arab Republic | 393 | 48% | 204 |
| Low | TCD | Chad | 667 | 58% | 280 |
| Low | TGO | Togo | 258 | 83% | 44 |
| Low | UGA | Uganda | 1581 | 91% | 142 |
| Low | YEM | Yemen, Rep. | 955 | 72% | 267 |
| Low | ZMB | Zambia | 626 | 91% | 56 |
| Lower middle | AGO | Angola | 1229 | 45% | 676 |
| Lower middle | BEN | Benin | 438 | 76% | 105 |
| Lower middle | BGD | Bangladesh | 2952 | 98% | 59 |
| Lower middle | BOL | Bolivia | 255 | 70% | 77 |
| Lower middle | BTN | Bhutan | 10 | 98% | 0 |
| Lower middle | CIV | Côte d’Ivoire | 853 | 76% | 205 |
| Lower middle | CMR | Cameroon | 885 | 69% | 274 |
| Lower middle | COG | Congo, Rep. | 171 | 77% | 39 |
| Lower middle | COM | Comoros | 23 | 85% | 3 |
| Lower middle | CPV | Cabo Verde | 10 | 93% | 1 |

| | | | | | |
|---|---|---|---|---|---|
| Lower middle | DJI | Djibouti | 23 | 59% | 10 |
| Lower middle | DZA | Algeria | 965 | 91% | 87 |
| Lower middle | EGY | Egypt, Arab Rep. | 2439 | 96% | 98 |
| Lower middle | FSM | Micronesia, Fed. Sts. | 2 | 72% | 1 |
| Lower middle | GHA | Ghana | 870 | 98% | 17 |
| Lower middle | HND | Honduras | 213 | 77% | 49 |
| Lower middle | HTI | Haiti | 257 | 51% | 126 |
| Lower middle | IDN | Indonesia | 4451 | 67% | 1469 |
| Lower middle | IND | India | 22687 | 85% | 3403 |
| Lower middle | IRN | Iran, Islamic Rep. | 1258 | 98% | 25 |
| Lower middle | KEN | Kenya | 1393 | 91% | 125 |
| Lower middle | KGZ | Kyrgyz Republic | 161 | 89% | 18 |
| Lower middle | KHM | Cambodia | 320 | 92% | 26 |
| Lower middle | KIR | Kiribati | 3 | 92% | 0 |
| Lower middle | LAO | Lao PDR | 158 | 75% | 39 |
| Lower middle | LBN | Lebanon | 83 | 67% | 27 |
| Lower middle | LKA | Sri Lanka | 308 | 96% | 12 |
| Lower middle | LSO | Lesotho | 55 | 87% | 7 |
| Lower middle | MAR | Morocco | 650 | 99% | 6 |
| Lower middle | MMR | Myanmar | 897 | 37% | 565 |
| Lower middle | MNG | Mongolia | 74 | 95% | 4 |
| Lower middle | MRT | Mauritania | 142 | 68% | 45 |
| Lower middle | NGA | Nigeria | 7130 | 56% | 3137 |
| Lower middle | NIC | Nicaragua | 139 | 87% | 18 |
| Lower middle | NPL | Nepal | 592 | 91% | 53 |
| Lower middle | PAK | Pakistan | 5974 | 83% | 1016 |
| Lower middle | PHL | Philippines | 2407 | 57% | 1035 |
| Lower middle | PNG | Papua New Guinea | 243 | 31% | 168 |
| Lower middle | PSE | West Bank and Gaza | 142 | 95% | 7 |
| Lower middle | SEN | Senegal | 524 | 85% | 79 |
| Lower middle | SLB | Solomon Islands | 20 | 87% | 3 |
| Lower middle | SLV | El Salvador | 101 | 79% | 21 |
| Lower middle | STP | São Tomé and Príncipe | 6 | 97% | 0 |
| Lower middle | SWZ | Eswatini | 28 | 77% | 6 |
| Lower middle | TJK | Tajikistan | 256 | 97% | 8 |
| Lower middle | TLS | Timor-Leste | 32 | 86% | 4 |
| Lower middle | TUN | Tunisia | 202 | 97% | 6 |
| Lower middle | TZA | Tanzania | 2157 | 81% | 410 |
| Lower middle | UKR | Ukraine | 344 | 78% | 76 |
| Lower middle | UZB | Uzbekistan | 806 | 98% | 16 |
| Lower middle | VEN | Venezuela, RB | 455 | 56% | 200 |
| Lower middle | VNM | Vietnam | 1469 | 83% | 250 |
| Lower middle | VUT | Vanuatu | 9 | 62% | 3 |
| Lower middle | WSM | Samoa | 6 | 85% | 1 |

| | | | | | |
|---|---|---|---|---|---|
| Lower middle | ZWE | Zimbabwe | 465 | 86% | 65 |
| Upper middle | ALB | Albania | 28 | 98% | 1 |
| Upper middle | ARG | Argentina | 642 | 76% | 154 |
| Upper middle | ARM | Armenia | 35 | 93% | 2 |
| Upper middle | AZE | Azerbaijan | 137 | 89% | 15 |
| Upper middle | BGR | Bulgaria | 60 | 89% | 7 |
| Upper middle | BIH | Bosnia and Herzegovina | 30 | 73% | 8 |
| Upper middle | BLR | Belarus | 90 | 98% | 2 |
| Upper middle | BLZ | Belize | 7 | 83% | 1 |
| Upper middle | BRA | Brazil | 2800 | 68% | 896 |
| Upper middle | BWA | Botswana | 60 | 95% | 3 |
| Upper middle | CHN | China | 13347 | 99% | 133 |
| Upper middle | COL | Colombia | 733 | 86% | 103 |
| Upper middle | CRI | Costa Rica | 63 | 99% | 1 |
| Upper middle | CUB | Cuba | 107 | 99% | 1 |
| Upper middle | DMA | Dominica | 1 | 92% | 0 |
| Upper middle | DOM | Dominican Republic | 202 | 84% | 32 |
| Upper middle | ECU | Ecuador | 298 | 72% | 83 |
| Upper middle | FJI | Fiji | 18 | 99% | 0 |
| Upper middle | GAB | Gabon | 61 | 75% | 15 |
| Upper middle | GEO | Georgia | 49 | 85% | 7 |
| Upper middle | GNQ | Equatorial Guinea | 47 | 53% | 22 |
| Upper middle | GRD | Grenada | 2 | 72% | 1 |
| Upper middle | GTM | Guatemala | 376 | 79% | 79 |
| Upper middle | GUY | Guyana | 16 | 91% | 1 |
| Upper middle | IRQ | Iraq | 1148 | 78% | 253 |
| Upper middle | JAM | Jamaica | 33 | 90% | 3 |
| Upper middle | JOR | Jordan | 240 | 77% | 55 |
| Upper middle | KAZ | Kazakhstan | 412 | 95% | 21 |
| Upper middle | LBY | Libya | 121 | 73% | 33 |
| Upper middle | LCA | St. Lucia | 2 | 80% | 0 |
| Upper middle | MDA | Moldova | 39 | 87% | 5 |
| Upper middle | MDV | Maldives | 8 | 96% | 0 |
| Upper middle | MEX | Mexico | 1938 | 78% | 426 |
| Upper middle | MHL | Marshall Islands | 1 | 86% | 0 |
| Upper middle | MKD | North Macedonia | 20 | 81% | 4 |
| Upper middle | MNE | Montenegro | 7 | 83% | 1 |
| Upper middle | MUS | Mauritius | 13 | 92% | 1 |
| Upper middle | MYS | Malaysia | 509 | 95% | 25 |
| Upper middle | NAM | Namibia | 67 | 93% | 5 |
| Upper middle | PER | Peru | 588 | 82% | 106 |
| Upper middle | PLW | Palau | 0 | 95% | 0 |
| Upper middle | PRY | Paraguay | 136 | 70% | 41 |
| Upper middle | RUS | Russian Federation | 1466 | 97% | 44 |

| | | | | | |
|---|---|---|---|---|---|
| Upper middle | SRB | Serbia | 69 | 92% | 6 |
| Upper middle | SUR | Suriname | 11 | 72% | 3 |
| Upper middle | THA | Thailand | 658 | 97% | 20 |
| Upper middle | TKM | Turkmenistan | 135 | 97% | 4 |
| Upper middle | TON | Tonga | 2 | 99% | 0 |
| Upper middle | TUR | Türkiye | 1270 | 95% | 63 |
| Upper middle | TUV | Tuvalu | 0 | 94% | 0 |
| Upper middle | VCT | St. Vincent and the Grenadines | 1 | 97% | 0 |
| Upper middle | ZAF | South Africa | 1185 | 86% | 166 |
| High | AND | Andorra | 0 | 99% | 0 |
| High | ARE | United Arab Emirates | 98 | 96% | 4 |
| High | ATG | Antigua and Barbuda | 1 | 92% | 0 |
| High | AUS | Australia | 304 | 95% | 15 |
| High | AUT | Austria | 84 | 85% | 13 |
| High | BEL | Belgium | 117 | 98% | 2 |
| High | BHR | Bahrain | 19 | 98% | 0 |
| High | BHS | Bahamas, The | 5 | 75% | 1 |
| High | BRB | Barbados | 3 | 82% | 1 |
| High | BRN | Brunei Darussalam | 6 | 99% | 0 |
| High | CAN | Canada | 375 | 92% | 30 |
| High | CHE | Switzerland | 85 | 96% | 3 |
| High | CHL | Chile | 229 | 95% | 11 |
| High | COK | Cook Islands | 0 | 98% | 0 |
| High | CYP | Cyprus | 13 | 96% | 1 |
| High | CZE | Czech Republic | 109 | 94% | 7 |
| High | DEU | Germany | 777 | 91% | 70 |
| High | DNK | Denmark | 62 | 97% | 2 |
| High | ESP | Spain | 354 | 92% | 28 |
| High | EST | Estonia | 14 | 90% | 1 |
| High | FIN | Finland | 46 | 89% | 5 |
| High | FRA | France | 667 | 96% | 27 |
| High | GBR | United Kingdom | 698 | 93% | 49 |
| High | GRC | Greece | 83 | 99% | 1 |
| High | HRV | Croatia | 36 | 92% | 3 |
| High | HUN | Hungary | 93 | 99% | 1 |
| High | IRL | Ireland | 60 | 94% | 4 |
| High | ISL | Iceland | 5 | 92% | 0 |
| High | ISR | Israel | 180 | 98% | 4 |
| High | ITA | Italy | 413 | 94% | 25 |
| High | JPN | Japan | 825 | 96% | 33 |
| High | KNA | St. Kitts and Nevis | 1 | 96% | 0 |
| High | KOR | Korea, Rep. | 302 | 98% | 6 |
| High | KWT | Kuwait | 50 | 94% | 3 |

| | | | | | |
|---|---|---|---|---|---|
| High | LTU | Lithuania | 27 | 90% | 3 |
| High | LUX | Luxembourg | 7 | 99% | 0 |
| High | LVA | Latvia | 18 | 94% | 1 |
| High | MCO | Monaco | 0 | 99% | 0 |
| High | MLT | Malta | 5 | 99% | 0 |
| High | NIU | Niue | 0 | 99% | 0 |
| High | NLD | Netherlands | 172 | 95% | 9 |
| High | NOR | Norway | 54 | 97% | 2 |
| High | NRU | Nauru | 0 | 98% | 0 |
| High | NZL | New Zealand | 62 | 90% | 6 |
| High | OMN | Oman | 87 | 99% | 1 |
| High | PAN | Panama | 76 | 74% | 20 |
| High | POL | Poland | 364 | 90% | 36 |
| High | PRT | Portugal | 86 | 99% | 1 |
| High | QAT | Qatar | 29 | 98% | 1 |
| High | ROU | Romania | 200 | 86% | 28 |
| High | SAU | Saudi Arabia | 647 | 97% | 19 |
| High | SGP | Singapore | 48 | 96% | 2 |
| High | SMR | San Marino | 0 | 90% | 0 |
| High | SVK | Slovak Republic | 57 | 97% | 2 |
| High | SVN | Slovenia | 20 | 86% | 3 |
| High | SWE | Sweden | 116 | 98% | 2 |
| High | SYC | Seychelles | 2 | 94% | 0 |
| High | TTO | Trinidad and Tobago | 18 | 94% | 1 |
| High | URY | Uruguay | 36 | 89% | 4 |
| High | USA | United States | 3735 | 93% | 261 |
| | | World weighted average | 132071 | 81.4% | 24568 |

Data source: WHO-UNICEF Estimates of National Immunization Coverage (WUENIC): 2021 revision
