# Supplementary material for: Estimating global and regional between-country inequality in routine childhood vaccine coverage in 195 countries and territories from 2019 to 2021: a longitudinal study
Source: eClinicalMedicine. 2023 Jun 8;60:102042. doi: 10.1016/j.eclinm.2023.102042 (PMC10249397; doi:10.1016/j.eclinm.2023.102042)
Supplement: Abstract in Chinese [file mmc2.pdf]

The following translations in Chinese were submitted by the authors and we reproduce them as supplied. They have not been peer reviewed. Our editorial processes have only been applied to the original abstract in English, which should serve as reference for this manuscript.

中文摘要

背景：近年全球常规儿童疫苗覆盖率趋于平稳，COVID-19 大流行进一步扰乱了免疫接种服务。本研究估计了 2019-2021 年全球和地区儿童常规疫苗覆盖率的不平等程度，特别是评估了 COVID-19 大流行的影响。

方法：我们使用了 WHO-UNICEF 国家免疫覆盖率估计（WUENIC）中 11 种常规儿童疫苗的接种纵向数据，覆盖 2019-2021 年的 195 个国家和地区。通过线性回归计算每种疫苗的不平等斜率指数（SII）和相对不平等指数（RII），以展示全球和地区层面最高和最低 20%的国家之间的覆盖率差异。我们还探讨了按 WHO 地区划分的儿童常规疫苗覆盖率的不平等性，以及按收入分组划分的未接种儿童数。

结果：在全球范围内，2019 年 1 月 1 日至 2021 年 12 月 31 日期间，绝大多数儿童疫苗的覆盖率呈下降趋势，未接种儿童数随之增加，特别是在低收入和中低收入国家。11 种常规儿童疫苗覆盖率指标均存在不平等现象。2019 年，第三剂 DTP 疫苗（DTP3）覆盖率的 SII 为 20.1 个百分点（95%置信区间：13.7，26.5），2020 和 2021 年分别上升至 23.6（17.5，30.0）和 26.9（20.0，33.8）。在 RII 指标和其他常规疫苗中也发现了类似结果。2021 年，第二剂麻疹疫苗（MCV2）覆盖率的全球绝对不平等程度最高（31.2，[21.5-40.8]），而轮状病毒疫苗（RotaC）覆盖率的不平等程度最低（7.8，[-3.9，19.5]）。在 WHO 的 6 个地区中，欧洲地区的不平等程度保持最低，而西太平洋地区在诸多指标中的不平等程度最高，尽管 2019-2021 年这两个地区的不平等程度都有所增加。

解释：全球和地区儿童常规疫苗覆盖率的不平等现象持续存在，且在 2019-2021 年大幅增加。研究揭示了在不同疫苗、地区和国家间，与经济发展水平相关的疫苗覆盖不平等现象，并强调了降低这类不平等程度的重要性。在 COVID-19 大流行期间，这些不平等现象有所扩大，导致在收入水平较差的国家中，疫苗覆盖率更低，未接种儿童数量更多。

基金项目：比尔及梅琳达·盖茨基金会

关键词：不平等；疫苗覆盖率；COVID-19 大流行；全球儿童健康
